# Temporal convolutional networks and data rebalancing for clinical length of stay and mortality prediction

**DOI:** 10.1038/s41598-022-25472-z

**Published:** 2022-12-08

**Authors:** Bryan P. Bednarski, Akash Deep Singh, Wenhao Zhang, William M. Jones, Arash Naeim, Ramin Ramezani

**Affiliations:** 1 Department of Electrical and Computer Engineering, University of California - Los Angeles, Los Angeles, CA USA; 2 Department of Computer Science, University of California - Los Angeles, Los Angeles, CA USA; 3 School of Medicine, University of California - Irvine, Irvine, CA USA; 4 Center for Smart Health, University of California - Los Angeles, Room 580, Engineering 6, 404 Westwood Plaza, Los Angeles, CA 90095 USA

**Keywords:** Computational science, Health care

## Abstract

It is critical for hospitals to accurately predict patient length of stay (LOS) and mortality in real-time. We evaluate temporal convolutional networks (TCNs) and data rebalancing methods to predict LOS and mortality. This is a retrospective cohort study utilizing the MIMIC-III database. The MIMIC-Extract pipeline processes 24 hour time-series clinical objective data for 23,944 unique patient records. TCN performance is compared to both baseline and state-of-the-art machine learning models including logistic regression, random forest, gated recurrent unit with decay (GRU-D). Models are evaluated for binary classification tasks (LOS > 3 days, LOS > 7 days, mortality in-hospital, and mortality in-ICU) with and without data rebalancing and analyzed for clinical runtime feasibility. Data is split temporally, and evaluations utilize tenfold cross-validation (stratified splits) followed by simulated prospective hold-out validation. In mortality tasks, TCN outperforms baselines in 6 of 8 metrics (area under receiver operating characteristic, area under precision-recall curve (AUPRC), and F-1 measure for in-hospital mortality; AUPRC, accuracy, and F-1 for in-ICU mortality). In LOS tasks, TCN performs competitively to the GRU-D (best in 6 of 8) and the random forest model (best in 2 of 8). Rebalancing improves predictive power across multiple methods and outcome ratios. The TCN offers strong performance in mortality classification and offers improved computational efficiency on GPU-enabled systems over popular RNN architectures. Dataset rebalancing can improve model predictive power in imbalanced learning. We conclude that temporal convolutional networks should be included in model searches for critical care outcome prediction systems.

## Introduction

Healthcare spending has reached astronomical levels in the United States to $3.8 trillion (2010), which is 17.7% of the U.S. GDP. It is expected to grow at a rate of 5.4% annually to reach $6.2 trillion by 2028^1,2^. Under burgeoning value-based care programs, in which the financial risk of care provision is shifted from payers to providers, hospital systems are motivated to adopt machine learning (ML) to help reduce the $1 trillion of annual waste in healthcare spending^3,4^. A primary use of ML is in decision support tools to streamline organizational inefficiencies and improve accuracy in challenging clinical decision-making applications^5,6^. This challenge is highlighted by the fact that 80,000 Americans die every year due to clinical diagnostic errors that result in-part from the system's inability to integrate data sources in decision-making^4^. Accurately predicting length of stay (LOS) and mortality likelihoods near the time of patient admission directly impacts care outcomes^7,8^, provider resource allocation^9,10^, and patient satisfaction^11,12^. We can expect improvement in these domains across health systems with improved predictive accuracy of ML models^7,13^.

Critical care outcome prediction is a core problem for health systems. Historically, multiple logistic regression models, such as APACHE^14^ and SAPS^15^, have been used to predict outcomes in critically ill patients; however, it has been shown that modern ML approaches outperform existing systems^16,17^. Complex clinical decision support settings are often defined as having multivariable inputs that are of mixed type (numerical and categorical) and time-series by nature^18,19^. While time-series is a traditionally difficult application domain in artificial intelligence (AI), the temporal convolutional network (TCN) offers an architecture that is uniquely suited for sequential input.

TCNs were first proposed by Lea et. al. in 2016^20^ and were largely popularized by their state-of-the-art performance in a wide range of applications (image classification, polyphonic music modeling, language modeling) as demonstrated by Bai et. al. in 2018^19^. Preceding TCN’s, a combination of convolutional neural networks (CNNs) (to capture spatial or locality-based relationships) along with recurrent neural network (RNN) blocks (to capture temporal relationships) were frequently used. However, the hierarchical architecture of TCNs can capture spatio-temporal information simultaneously with a high degree of parallelism, making them favorable in the applications of graphics processing unit (GPU) to AI applications^19–24^. TCNs have recently found use in clinical applications such as early prediction of adverse events^25^, length of stay prediction^26^, and injury detection^27^. Catling and colleagues used TCNs to develop risk-prediction models which either perform comparably or outperform long short-term memory (LSTM) recurrent neural networks (RNN) in prediction of clinical events when provided one hour of temporal data^25^. Rocheteau and colleagues presented a similar temporal pointwise convolution model, which demonstrates performance benefits over LSTM and transformer models in ICU length of stay regression in MIMIC with additional model explainability analysis^26^.

Irrespective of the model, predictive performance of classifiers can be unsatisfactory with imbalanced datasets for which classes are not equally represented^28,29^. Inherent bias towards the majority class, known as class imbalance, may result in low accuracy when labeling minority classes^30,31^. This occurs because machine learning classifiers are often designed to minimize loss functions to maximize overall accuracy, which alone may not be satisfactory in application^32^. For instance, if the minority class makes up just 1% of the dataset, predicting every data point as belonging to the majority class will lead to a 99% accuracy—which many practitioners may initially interpret as satisfactory, even though the model did not learn.

Existing data rebalancing methods can be categorized into two classes: data-level and algorithm-level approaches.

Data-level rebalancing approaches manipulate the number of samples from either the outcome majority or minority to achieve a target ratio by either removing existing samples, duplicating existing samples, or generating synthetic data. Undersampling techniques remove random samples from the majority class, leaving all minority samples in place to achieve a desired outcome ratio^32,33^. Conversely, oversampling techniques duplicate or synthesize (with information theoretic algorithms) new data points for the under-represented class to achieve a target ratio. There are numerous synthetic oversampling techniques presented in the literature, including the Synthetic Minority Oversampling Technique (SMOTE)^29,30^ and the adaptive synthetic (ADASYN)^34^ classes of solutions. This work focuses on evaluating multiple SMOTE methods.

In algorithm-level rebalancing, the reweighting of minority and majority classes is performed directly within the model rather than during data preprocessing and can be further grouped into cost-sensitive learning and ensemble learning. Cost-sensitive learning methods penalize more for the misclassifications of the minority class in the loss function^35,36^. Ensemble learning methods train a series of machine learning models (subtasks) and the prediction outcome from each model constitutes the overall predictive decision, aggregated via a weighted voting method. SMOTEBoost and RUSBoot are examples of ensemble rebalancing methods^30,37^.

### Significance

In this study, we utilize the PhysioNet MIMIC-III critical care dataset to evaluate how well TCNs can predict patient LOS and mortality from strictly time series input data^9,38–41^. By extending a core data processing pipeline and evaluating state-of-the-art deep learning models to modern medical informatics standards, we make the following contributions:

- Improve established MIMIC-III preprocessing pipeline so that we may evaluate ML models in a simulated prospective study with rigorous cross-validation for hyperparameter selection and unseen hold-out validation.
- Evaluate and justify the temporal convolutional networks (TCN) for critical care prediction model architecture searches.
- Demonstrate the novel application of training data rebalancing (both non-synthetic and synthetic methods) for TCNs and analyze the influence of modern rebalancing algorithms on outcome prediction performance.
- Display the benefits of including the TCN in optimal model searches for critical care outcome prediction tasks.

## Materials and methods

The authors of this manuscript have made the code for the model and validation pipeline available on GitHub (https://github.com/bbednarski9/MIMICIII_TCN) under the MIT License.

### Source data

The Medical Information Mart for Intensive Care (MIMIC-III Clinical Database v.1.4) makes available for research the de-identified (in accordance with Health Insurance Portability and Accountability Act [HIPAA]) medical records of 53,423 patients from the Beth Israel Deaconess Medical Center (Boston, MA) between 2001 and 2012^38–40^. Patients in this study database were provided informed consent and data collection complied with the Declaration of Helsinki. Authors have been approved for ethical data use and credentialed access to the publicly available MIMIC-III dataset for data analysis and model development by the managing group: Laboratory for Computational Physiology at Massachusetts Institute of Technology per the PhysioNet Credentialed Health Data License 1.5.0, with whom this project is registered. Original details on data de-identification and public credentialed access are provided in^38^.

The MIMIC-Extract preprocessing pipeline filters to admissions in which patients were admitted to the ICU for the first time, were over 15 years of age, and the length of stay is at least 10 hours and fewer than 10 days^41^. Under these rigorous criteria, the resultant cohort consists of 23,944 patient records (56% male; median age: 66, interquartile range [IQR]: 53–78; median length of stay: 2.7 days, IQR: 1.9–4.2) which can be used for evaluation of length of stay and mortality classification tasks. To evaluate our model, and rigorously re-evaluate baseline models presented in^41^, the data set is split 80/20%, utilizing the larger cohort for cross-validation and smaller cohort for simulated prospective hold-out testing. First, k-fold (k = 10) cross-validation (18,880 records) is used to identify the best hyperparameters and to train the model for hold-out validation. Within each fold data is split into tenths, utilizing 80% for model training, 10% for validation, and 10% for testing. The model with the best performance across all 10 folds is selected and applied directly to the hold-out set (5,064 records) for a robust final evaluation.

Constraining decision support data to real-time applications precludes the use of ICD procedure and diagnosis codes, which become available to health practitioners days or weeks after discharge^42–45^. Additionally, we exclude static demographic, clinical, and admission variables in this study. Though these static variables are often found to be strong risk predictors, they frequently result in model bias towards race, gender, and socioeconomic status due to their a priori distributions within clinical cohorts^46,47^. For example, if patients of color or lower socioeconomic status are more likely to be discharged early, a biased model could learn those associations and under-predict risk in similar patients. While the lower-bound for all model performances in this paper could be raised by including these variables, we instead elected to evaluate strictly for the predictive power from time-series vital signs data without bias.

Our dataset is filtered to strictly time-series vital signs data. Each patient record contains 312 clinical objective features for the first 24 hours of admission, totaling 7,488 features. The 312 features per hour consist of 104 clinical objective measurements with corresponding points for the number of hours since measurement and a mask identifying whether the value is measured at each hour. Ultimately, we classify this dataset as having a low sample to feature ratio (~ 3.2:1). Practitioners typically aim for a ratio between 5:1 (for slightly uncorrelated features) and 10:1 (for totally uncorrelated features)^48^.

### Clinical outcomes and variables

We evaluate the predictive accuracy of the TCN across four binary classification outcomes: LOS > 3 days, LOS > 7 days, hospital mortality, and ICU mortality. These outcomes were selected due to their low-complexity (for generalizability across health systems) and for our evaluation pipeline to be a direct extension of the simpler train/validation/test-split procedure demonstrated in the original MIMIC-Extract pipeline^41^. The national average length of stay is 4.7 days^14^, so the prediction of LOS > 3 and LOS > 7 can have a valuable impact in care coordination.

### The temporal convolution network (TCN) architecture

**Figure 1:**
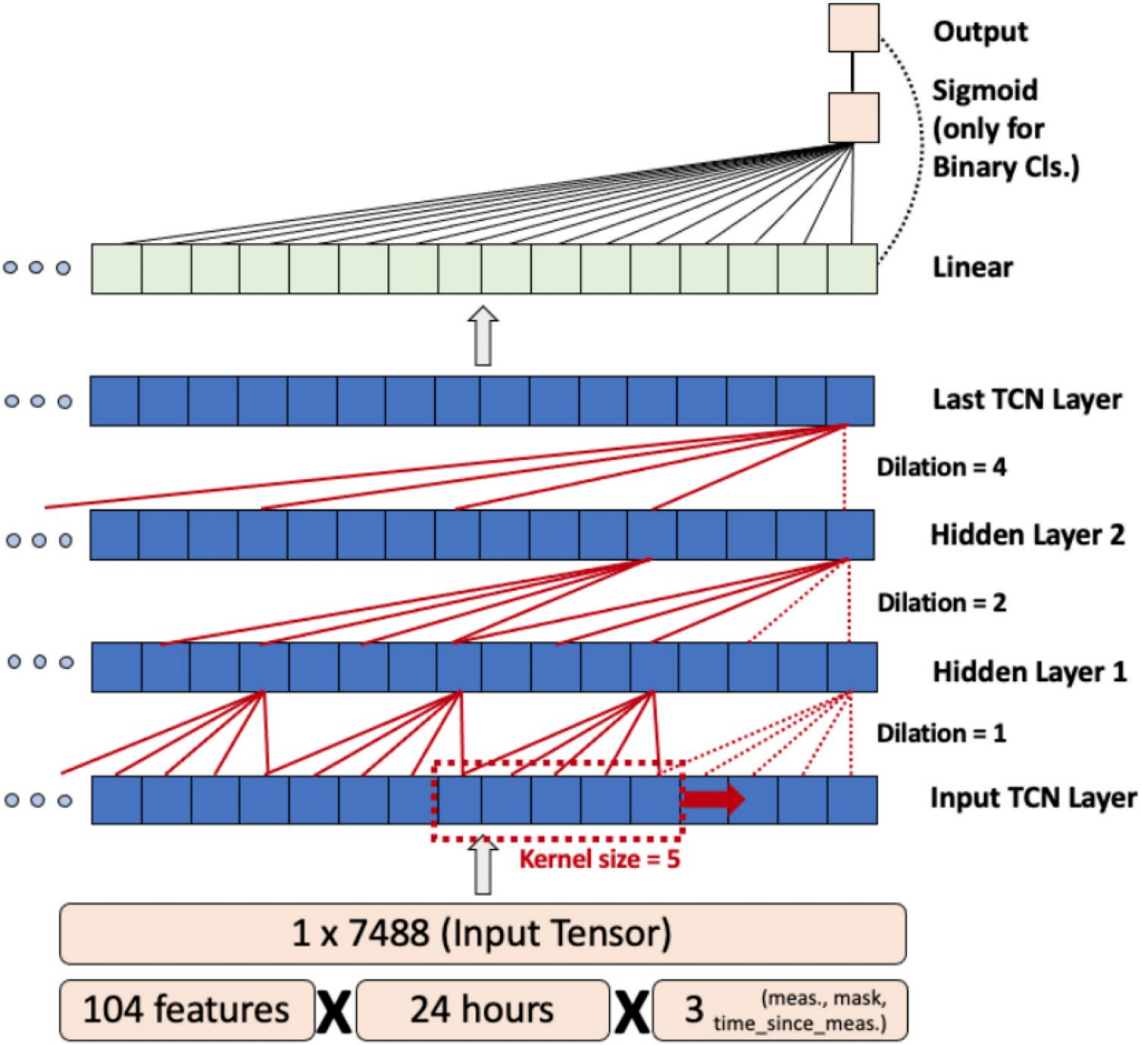
The temporal convolutional network (TCN) demonstrates a flexible input size due to its hierarchical architecture and exponential convolution dilation factor.

Figure 1 depicts a functional block diagram of the TCN. Given an input vector ***X***_***tf***_** = [*x***_**1**_***,…,x***_***tf***_**]** where ***t*** represents the length of the time series in hours, and ***f*** represents the number of features per hour. The TCN outputs ***Y***_***j***_** = [*y***_**1**_***,…,y***_***j***_**]** where ***j*** represents the length of the projected output sequence (j = n for BC with n classes, j = 1 for regression). TCNs exploit causal, dilated 1-D convolutions to learn long-term relationships between sequential inputs by sliding a 1-D kernel (of length ***k***) across the input sequence (***X***_***tf***_) while normalizing the output into the subsequent layer of the model^19–24^. We use a fixed exponential dilation factor of ***b* = 2**, where at the ***ith*** layer, the intermediate dilation factor ***d***_***i***_** = *b***^***i***^, and the kernel would skip over ***b***^***i***^**−1** values between computations. Additionally, a residual block connection has been added between every other layer to prevent overfitting^22^. The input receptive field (***w***) of a TCN is dependent on three parameters: convolutional kernel size (***k***), the number of hidden layers (***n***), and the dilation factor (**b**), computed as shown in Eq. 1. Exponential growth of **w** with the dilation factor **b** allows TCNs to function with large receptive fields.

1 $$w = 1 + \left( {k - 1} \right) \cdot \frac{{b^{n} - 1}}{b - 1}$$

Computing maximum receptive field (w) for the TCN network given hidden layers (n), convolutional kernel size (k), dilation factor (b).

### Baseline models

For performance context, multivariable logistic regression (LR), random forest (RF), and gated recurrent unit with decay (GRU-D) models are also evaluated. Both LR and RF are well-established in medical informatics literature^15,16^. The GRU-D model is a recurrent neural network (RNN) architecture (similar to the long short-term memory network [LSTM])^49^. GRU-D was selected over LSTM because it was demonstrated as a state-of-the-art for this dataset in^41^ and to outperform LSTM for MIMIC data^50^. TCN has already been demonstrated to outperform LSTM in^25^.

### Evaluation metrics

Model hyperparameters are selected during cross-validation and performance is computed with aggregate predictions from all folds. The best performing model from all folds is determined (by average area under receiver operating characteristic [AUROC]), retrained on all available data, and validated on the unseen hold-out set. Models are compared in terms of AUROC, area under precision-recall curve (AUPRC), accuracy, and F-1 measure. Precision and recall are included to indicate driving factors for the F-1 score (harmonic mean). AUROC and AUPRC are evaluated across all predictive thresholds (0 to 1.0). AUROC evaluates a model’s discriminative capability by comparing the true positive rate (TPR) and false positive rate (FPR)^51^. AUPRC is considered as better evaluation metric for imbalanced datasets compared to AUROC, as it directly includes false-positive (FP) and false-negative (FN) predictions in its evaluation^52^. Accuracy, precision, recall, and F-1 are evaluated at activation threshold of *p* = 0.5. Accuracy is shown as a baseline for predictive performance. Brier scores quantify model calibration. Bootstrapping (1000 iterations) is performed to provide 95% confidence intervals (CI) for all outcome evaluation metrics.

### Data rebalancing

We evaluate the performance of rebalancing algorithms similarly to both cross-validation and hold-out validation for direct comparison to the original non-rebalanced experiments. For LOS > 3, a largely balanced task, only re-sampling to an outcome distribution ratio of 1:1 was feasible. However, for largely unbalanced tasks (LOS > 7, In-Hospital Mortality, In-ICU Mortality), data was rebalanced to ratios of 1:1, 1:2, 1:3, 1:4, and 1:5. Methods compared across each BC task include:

- Random (majority) under-sampling (no synthetic data)^53^
- Random (minority) over-sampling (duplicate data)^54^
- Synthetic Minority Oversampling Technique (SMOTE) (synthetic data)^30,54^
- Borderline (BL) SMOTE (synthetic data)^54,55^
- Support vector machine (SVM) SMOTE (synthetic data)^54,56^

## Results

### Performance of TCN in binary classification

The distribution of outcome events across cross-validation and hold-out validation splits is provided in Table 1.

**Table 1:** Inner-task event frequency is consistent between cross-validation and hold-out validation cohorts for all four binary classification outcomes (0.05–0.84%). Intra-task event frequency provides diversity between binary classification outcomes (7.17–43.03%).

Split	Patient total	LOS > 3		LOS > 7		ICU mortality		Hospital mortality	
		n	%	n	%	n	%	n	%
Cross-validation (tenfold)	18,880	8,126	43.0	1446	7.7	2037	10.8	1369	7.3
Hold-out	5,064	2,177	43.0	399	7.9	504	10.0	348	6.9
Total	23,944	10,303	43.0	1845	7.7	2541	10.6	1717	7.2

LOS, length of stay.

Table 2 presents the performance of all models for all four BC tasks, validated with the hold-out set. Overall, the TCN demonstrates best performance in 6 of 16 evaluation metrics (four metrics across four tasks: AUROC, AUPRC, Accuracy, F-1 measure), while the GRU-D model demonstrates best performance in 9 of 16.

**Table 2:** Hold-out validation performance of all models in all binary classification tasks (value ± 95% CI).

Model	AUROC	AUPRC	Accuracy	F-1	Precision	Recall
**In-ICU mortality**						
LR	85.1 ± 3.2	39.5 ± 7.2	93.4 ± 0.6	30.1 ± 7.6	55.0 ± 11.6	20.7 ± 6.1
RF	89.1 ± 2.2	45.9 ± 7.3	93.5 ± 0.3	14.2 ± 6.5	81.8 ± 19.2	7.8 ± 3.9
GRU-D	**89.4 ± 2.3**	**50.8 ± 6.8**	94.0 ± 0.6	38.9 ± 8.1	66.2 ± 10.3	27.6 ± 6.5
TCN	89.2 ± 2.5	**50.8 ± 7.0**	**94.3 ± 0.6**	**46.6 ± 7.3**	64.5 ± 8.7	36.5 ± 7.1
**In-hospital mortality**						
LR	83.6 ± 2.6	44.7 ± 5.7	91.0 ± 0.7	35.7 ± 6.0	61.4 ± 9.3	25.2 ± 5.3
RF	86.4 ± 2.3	49.3 ± 5.9	90.7 ± 0.4	14.5 ± 5.8	85.1 ± 14.0	7.9 ± 3.4
GRU-D	87.3 ± 2.3	52.1 ± 5.6	**91.6 ± 0.8**	44.2 ± 6.0	65.4 ± 7.5	33.4 ± 5.8
TCN	**87.7 ± 2.1**	**53.0 ± 6.0**	91.2 ± 0.9	**47.2 ± 6.0**	58.7 ± 6.7	39.5 ± 6.2
**Length of stay (LOS > 3)**						
LR	69.0 ± 2.1	61.7 ± 2.8	65.5 ± 1.8	53.5 ± 2.7	63.6 ± 2.8	46.2 ± 2.9
RF	71.4 ± 2.0	65.5 ± 2.8	67.3 ± 1.7	55.3 ± 2.7	67.1 ± 2.8	47.0 ± 3.0
GRU-D	**72.2 ± 2.0**	**65.7 ± 2.7**	**68.1 ± 1.7**	**59.4 ± 2.5**	65.6 ± 2.6	54.2 ± 3.0
TCN	71.6 ± 2.2	65.0 ± 2.7	67.0 ± 1.7	55.6 ± 2.7	66.0 ± 2.8	48.0 ± 2.9
**Length of stay (LOS > 7)**						
LR	66.8 ± 4.2	15.9 ± 3.3	91.7 ± 0.3	2.3 ± 2.8	15.2 ± 17.7	1.3 ± 1.6
RF	**75.3 ± 3.5**	22.0 ± 4.5	**92.1 ± 0.0**	0.0 ± 0.0	0.0 ± 0.0	0.0 ± 0.0
GRU-D	74.4 ± 3.8	**22.4 ± 4.5**	92.0 ± 0.4	**9.8 ± 5.3**	44.9 ± 20.4	5.5 ± 3.2
TCN	73.5 ± 3.6	18.8 ± 3.5	91.8 ± 0.3	3.7 ± 3.5	25.0 ± 21.9	2.0 ± 1.9

All values shown in %. Primary evaluation metrics: AUROC, AUPRC, Accuracy, F-1. Secondary evaluation metrics: precision, recall. TCN, temporal convolution network; GRU-D, gated recurrent unit with delay; RF, random forest; LR, logistic regression; AUROC, area under receiver operating curve; AUPRC, area under precision recall curve.

Best-in-task values for primary evaluation metrics are in bold.

For mortality prediction tasks (in-ICU, in-Hospital), we observe that the TCN outperforms other models in 6 of 8 critical metrics. For these tasks, the deep learning models (TCN, GRU-D) demonstrate the best performance in all metrics. For AUROC, AUPRC and accuracy, the difference between TCN and GRU performance is < 1.0%. However, in F-1 measure the TCN outperforms the GRU-D (ICU: + 7.7%, Hospital: + 3.0%).

In both length of stay tasks (LOS > 3, LOS > 7), the GRU-D is the best performer in 6 of 8 metrics, while the random forest classifier performs best in AUROC and accuracy for LOS > 3. For each of these task-metric pairs, performance of the TCN falls behind the GRU-D between 0.2 and 6.1%. Supplementary Materials A presents the performance of all models for all four BC tasks, evaluated on the cross-validation classification results.

We observe that F-1 measure scores for all four models are low for the LOS > 7 task. Lower recall than precision scores indicate that for this task, all models are generally over-predicting false negatives.

### Model calibration

To compare the default probabilistic accuracy (calibration) of all four models, we present Brier scores for each task and validation procedure. Results for both cross-validation and hold-out validation are found in Table 3. Graphical depictions are found in Supplementary Figures 1–8. The largest difference for inner-task scores was 1.2%. The largest difference between the TCN and other best-in-task models was only 0.3%, suggesting similar probabilistic accuracy and non-inferiority of the TCN compared to proven models.

**Table 3:** Model calibration comparison using Brier scores for both hold-out validation and cross-validation. Inner-task comparison demonstrates similar (or stronger) calibration of TCN to baseline models.

Model	LOS > 3	LOS > 7	ICU mortality	Hospital mortality
**Hold-out test calibration results (Brier score)**				
LR	0.221	0.072	0.052	0.071
RF	0.211	**0.067**	0.049	0.069
GRU-D	**0.209**	0.070	**0.046**	**0.066**
TCN	0.211	0.070	0.047	0.067
**Cross-validation calibration results (Brier score)**				
LR	0.220	0.071	0.055	0.077
RF	0.207	0.066	0.052	0.075
GRU-D	**0.206**	0.069	**0.050**	0.070
TCN	0.209	**0.067**	**0.050**	**0.069**

TCN, temporal convolution network; GRU-D, gated recurrent unit with delay; RF, random forest; LR, logistic regression; LOS, length of stay.

Best-in-task values are in bold.

### Dataset rebalancing

**Figure 2:**
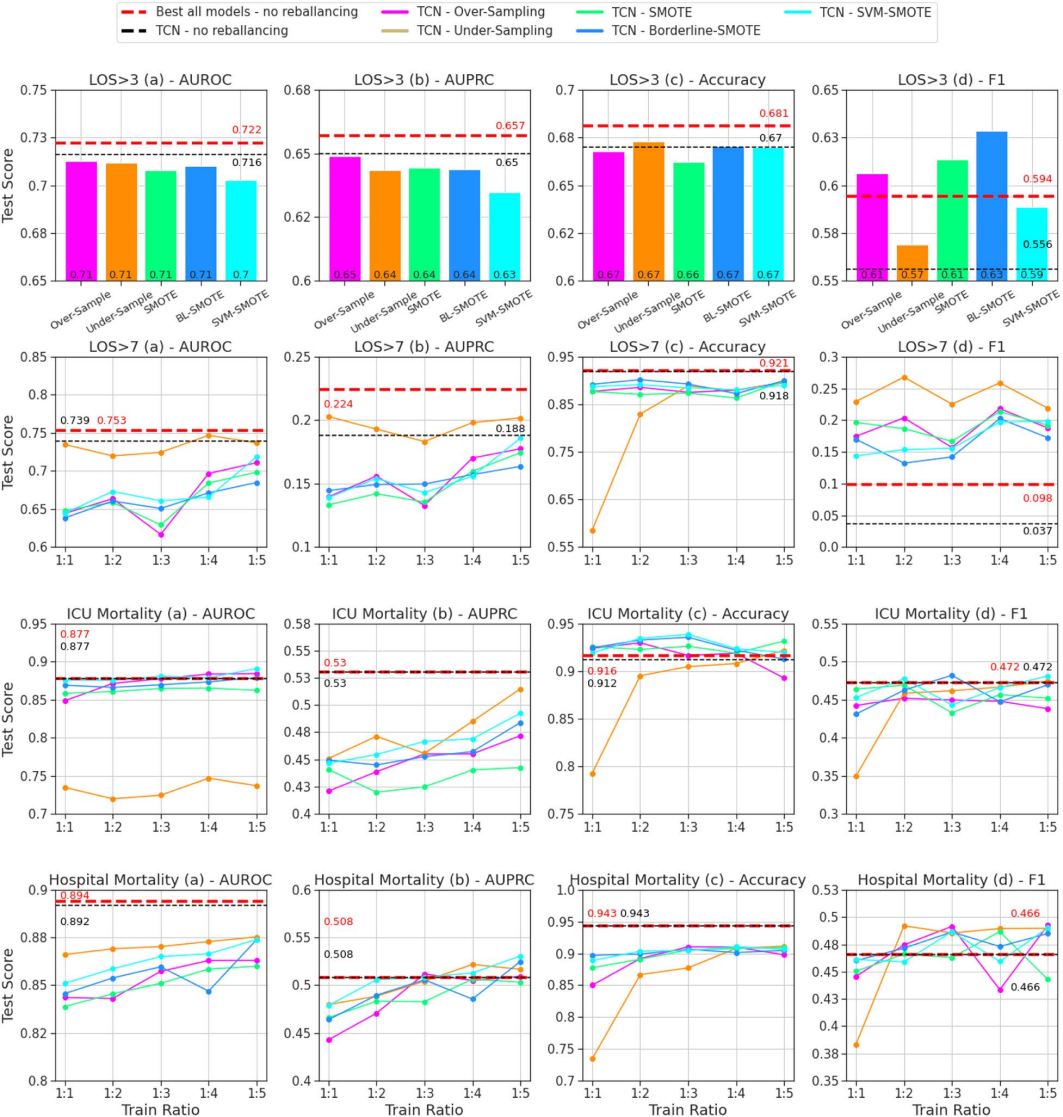
Evaluation of the TCN model with all rebalancing methods for hold-out validation cohort. Binary classification tasks (4) ordered by rows; evaluation metrics (4) ordered by columns. Dashed lines (red/black) represent TCN and best-in-task (all models) performance without rebalancing. For the three tasks where possible (LOS > 7, ICU mortality, hospital mortality), methods are evaluated for rebalancing ratios of 1:1, 1:2, 1:3, 1:4, and 1:5, otherwise only 1:1. TCN, temporal convolutional network; AUROC, area under the receiver-operating characteristics; AUPRC, area under the precision-recall curve; LOS, length of stay; BL, borderline; SVM, support vector machine; SMOTE, synthetic minority oversampling technique.

The performance of rebalancing methods and ratios for the best TCN model from each BC task on the hold-out validation set are summarized in Fig. 2, with direct comparison to the baseline TCN without rebalancing (black dashed lines) and best overall model for each task without rebalancing (red dashed lined) from Table 1.

We compare rebalancing results for each task and metric to the TCN without rebalancing and observe that performance is improved by at least one rebalancing method and ratio in 10 of 16 cases. We compare rebalancing results to the best from all four baselines without rebalancing and observe that performance is improved in 8 of 16 cases. For LOS > 7, under-sampling to any ratio (1:1, 1:2, 1:3, 1:4, 1:5) significantly improves TCN performance in terms of F-1 score (+ 18.2 to + 23.1%) with minimal degradation in terms of AUROC (− 1.5 to + 1.2%) and AUPROC (− 0.5 to + 2.0%). The improvement of F-1 for LOS > 7 (Fig. 2) with rebalancing is notable because it was the worst task-metric pair in the original hold-out validation (Table 1). While poor performance without rebalancing was attributed to excessive false negative samples, we observe consistent improvement in recall for this outcome after rebalancing training data (Supplementary Table 2).

While performance for some tasks and metrics consistently improves with rebalancing, this is not observed in all circumstances. Performance degradation is observed for all methods and ratios in terms of AUROC for both LOS > 3 and hospital mortality, AUROC for ICU mortality and LOS > 3, and accuracy for LOS > 7 and hospital mortality prediction.

Complete rebalancing results for both cross-validation and hold-out validation are provided in Supplementary Materials C.

### Computational efficiency

**Figure 3:**
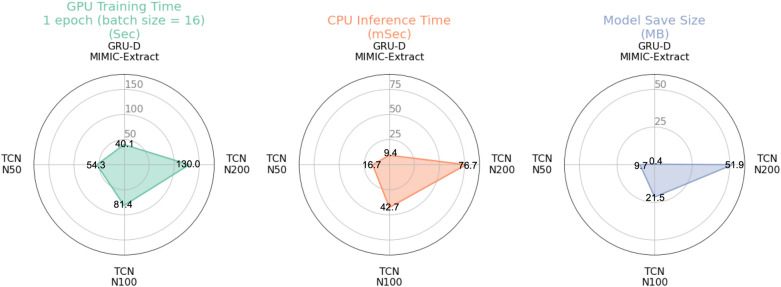
Comparing the computational complexity of the advanced baseline GRU-D model to three different TCN configurations. TCNs generally require less training time per model parameter than the GRU-D on GPU-based systems and demonstrate sub-second single-patient inference runtimes. GRU-D, gated recurrent unit with decay; TCN, temporal convolutional network; N, kernel density in TCN.

We compare the computational efficiency of deep learning models (TCN and GRU-D) on the same system (CPU: Intel i7-7700K 8-core [Intel Corporation, Santa Clara, CA]; GPU: NVIDIA 1080 [Nvidia Corporation, Santa Clara, CA]). Model runtime performance in terms of single-epoch GPU training time, single-patient CPU inference time, and model disk space are provided for a range of TCN and GRU-D models in Fig. 3. While our largest version of the TCN (layers = 12, kernel density = 200, kernel size = 5) requires 141 times greater the disk space to save compared to the baseline GRU-D (51.9 vs. 0.362 MB) the single epoch training time (single cross-validation fold) using a GPU with batch size 16 for the TCN, is only 3.2 times longer (130 vs. 40.13 s). Furthermore, the CPU inference time (presented as GPUs are typically absent from clinical deployment settings) for the TCN is only 76.7 ms compared to 9.43 ms for the GRU-D (8.1 times), and clearly tractable for real-time deployment. This comparison highlights the improved parallelization of the TCN architecture compared to the GRU-D on a GPU-enabled system. TCN hyperparameters are provided in Supplementary Table 4.

## Discussion

The primary aim of this study is to evaluate the predictive power of TCNs in critical care outcome prediction using the MIMIC-III dataset and MIMIC-Extract preprocessing pipeline^38–41^, and to compare this performance to high performance ML baselines. First, we demonstrated that the TCN efficiently learns to predict clinical outcomes in strictly time-series LOS and mortality classification tasks despite a priori varying inter-task outcome label distributions. We then verified with Brier scores that the default TCN was calibrated similarly to the advanced baseline (GRU-D) model. Next, we presented the performance of leading training data rebalancing methods and showed that they consistently improve TCN performance in terms of F-1 measure, and can potentially improve AUROC, AUPRC, and accuracy under rebalancing algorithms and outcome ratios. Lastly, we present key computational efficiency statistics for TCNs and analyze their implications to future clinical systems.

While model performance in this paper could be improved by including static clinical variables, we exclude these variables to reduce risk of model bias which could violate equity, diversity, and inclusion principles. It is important that model performance during development represents the core nature of the dataset—strictly time-series vital signs in this case. Yet still, the multi-modal nature of clinical data and standard practices in application may require the future integration of these variables. Catling and Wolff^25^ approach this problem with a separate fully connected branch and downstream layer concatenation. Rocheteau et al.^26^ approach this problem with a two-stream architecture. However, Fukuia et al.^57^ and Deng et al.^21^ point out that these methods are likely suboptimal as they do not leverage the interaction between weights and features at each network layer. The TCN allows downstream interactions between all input feature weights. Therefore, clinical inputs could be appended to the beginning of the temporal input to the TCN, allowing downstream interactions with all data passed to the model.

Imbalanced class label distributions are common in clinical applications^31,58^. Our rebalancing analysis demonstrates that a variety of methods and ratios can lead to significant model prediction improvement in terms of F-1 measure^29,32–37,59–61^. This is notable because it supports that data rebalancing can be used to improve the balance of FPs and FNs at a probability threshold of 0.5. This supports the use of rebalancing methods for tasks that seek to have equal weight for FNs and FPs to minimize total absolute error. We also observed improvement for AUROC (LOS > 7, ICU mortality), AUPRC (LOS > 7, hospital mortality), and accuracy (LOS > 3, ICU mortality) with select methods and ratios, demonstrating that rebalancing can improve general predictive performance. The degradation of AUPRC in some cases (see ICU mortality performance in Fig. 2) shows that the benefit to rebalancing may not hold across all thresholds (for all ratios and methods) and should not be applied naïvely.

As the applications for AI in medicine expand to diverse tasks^25–27^, system architects are increasingly responsible for comprehensive model architecture searches to identify optimal methods. Prior to clinical deployment it is imperative for practitioners to explore model explainability, interpretability, and feature importance methods. This understanding will allow for in-depth clinical analyses of model predictions and the reduction of unnecessary input parameters without compromising performance^62^. Unlike random forest ensemble models (like the popular XGBoost algorithm^47^), which have built-in interpretability, deep learning models are not equipped with feature importance scores by default. However, there are multiple off-the-shelf algorithms designed to extend AI algorithms such as SHAP^63,64^ or the integrated gradients method^65^. Rocheteau et. al. demonstrate that these methods are compatible with the TPC/TCN model architecture and are useful for clinical phenotyping and feature reduction before deployment^26^.

Our results support that TCNs are viable models for clinical decision support systems that are required to run in real-time with time series input data. They are highly parallelizable, have a flexible receptive field allowing for exponential input sequence size scaling (a variable dilation factor) and have low memory requirements during training. Conversely, RNN-based architectures (like the GRU-D) must be sequentially evaluated and demonstrate poor compute efficiency per parameter^66–68^. Systems equipped with basic GPU compute capabilities can efficiently prototype, train, and evaluate TCNs^19–24^. Larger memory requirements during training are a shortcoming of TCNs, causing them to also be less efficient to train on CPU-only systems. Regardless, we demonstrated that after training is complete, TCNs can evaluate single predictions efficiently enough for real-time deployment on CPU-only systems.

### Limitations

While the TCN offers somewhat improved performance over baselines in mortality prediction, its performance was lower than expected in LOS tasks and over all the TCN only outperforms the GRU-D by AUROC for in-hospital mortality. In general, the TCN and GRU-D have largely similar performances. However, the GRU-D was originally selected by database designers^41^ as a high performing AI baseline, so small predictive power differences between these models is not surprising.

Another limitation is the low sample-to-feature dimension ratio of this data^36^. We observed some signs of overfitting during model training which was counteracted using early stopping methods. A higher ratio of samples to features would likely diminish these issues, though early stopping is commonly applied and trusted in practice. A large input feature dimension significant obstacle for many temporal machine learning problems. Observations here help to justify future work in temporal data structure feature reduction.

The TCN was evaluated exclusively with time-series vital signs data. Many electronic medical record integrated systems such as APACHE^14^ and SAPS^15^ historically utilize numerous static variables, so a direct comparison was not within scope. However, multiple studies have already demonstrated superiority of modern machine learning algorithms to these models^16,17^.

Finally, it is important to note that the TCN’s computational efficiency during training is largely dependent on having a GPU-based system available. While GPUs have become commonplace in AI development settings, designers for applications in CPU-only domains should consider these runtime implications.

## Conclusions

The TCN model was rigorously evaluated in a simulated prospective study using the widely available MIMIC-III dataset for both LOS and mortality prediction. In some circumstances, such as mortality prediction, performance was improved over the state-of-the-art. We have also investigated dataset rebalancing as a method to improve model calibration and performance when the TCN was inferior to baselines. A complete evaluation of data rebalancing methods with the TCN is relevant to clinical predictions where class imbalance is common. Robust performance of the TCN when trained with strictly time series data emphasizes that the model is suitable for clinical systems where vital signs data is available and important to consider.

As the variety and size of deep learning models has generally increased in recent years, it has become more important than ever for practitioners to understand the situational implications of applying each. To this effect we have analyzed the implications of the TCN architecture in clinical applications, which allows for more efficient per-parameter training on GPU-enabled systems compared to popular RNN-based architectures. For these reasons, we believe that the TCN should be included in model searches for the next generation of AI clinical decision support systems.

## Supplementary Information

Supplementary Information.

## Data Availability

The MIMIC-III dataset used in this project was made freely available with credentialed access to the Physionet repository (http://www.physionet.org) as maintained by the MIT Laboratory for Computational Physiology. This dataset is available upon request at http://dx.doi.org/10.13026/C2XW26.
