# Supplementary material for: Temporal convolutional networks and data rebalancing for clinical length of stay and mortality prediction
Source: Sci Rep. 2022 Dec 8;12:21247. doi: 10.1038/s41598-022-25472-z (PMC9732283; doi:10.1038/s41598-022-25472-z)
Supplement: Supplementary file 1 — Supplementary Information. [file 41598_2022_25472_MOESM1_ESM.docx]

**Temporal Convolutional Networks and Data Rebalancing for Clinical Length of Stay and Mortality Prediction**

**Supplementary Materials A - D**

- **Supplementary Materials A** – Cross-Validation Classification Results
  - Begins: Pg. 2; Contains: **Supplementary Table 1**

**Supplementary Materials B** – Brier Score Calibration Plots

- - Begins: Pg. 3: Contains: **Supplementary Figure 1 – Supplementary Figure 8**
- **Supplementary Materials C** –Rebalancing Cross-Validation/Hold-Out Results
  - Begins: Pg. 5; Contains: **Supplementary Table 2, Supplementary Table 3, Supplementary Figure 9**
- **Supplementary Materials D** – TCN Hyperparameters
  - Begins: Pg. 12; Contains: **Supplementary Table 4**

* Correspondence to: Bryan Bednarski, bryanbed@g.ucla.edu, +1 (626) 399-2803

Center for Smart Health, Room 580, Engineering 6, 404 Westwood Plaza, Los Angeles, CA, 90095

**Supplementary Appendix A – Cross-Validation Classification Results**

**Supplementary Table 1:** 10-fold cross-validation performance of all models in all binary classification tasks (value +/- 95% CI). TCN: temporal convolution network, GRU-D: gated recurrent unit with delay, RF: random forest, LR: logistic regression.

| **Length of Stay (LOS>3)** | | | | | | | |
| --- | --- | --- | --- | --- | --- | --- | --- |
| **Model** | **Task** | **AUROC** | **AUPRC** | **ACCURACY** | **PRECISION** | **RECALL** | **F1** |
| LR | LOS>3 | 0.691014 (0.010680) | 0.615587 (0.013862) | 0.663665 (0.008990) | 0.648545 (0.014353) | 0.477111 (0.015683) | 0.549773 (0.013551) |
| RF | LOS>3 | 0.726452 (0.010355) | 0.674025 (0.014242) | 0.684269 (0.008974) | 0.690146 (0.014572) | 0.483510 (0.015159) | 0.568637 (0.013841) |
| GRU-D | LOS>3 | **0.729915 (0.010146)** | **0.681426 (0.013544)** | **0.690837 (0.008618)** | **0.688024 (0.014121)** | **0.515383 (0.015357)** | **0.589320 (0.012894)** |
| TCN | LOS>3 | 0.722935 (0.010514) | 0.660374 (0.014704) | 0.680636 (0.008822) | 0.684113 (0.015018) | 0.479518 (0.015951) | 0.563829 (0.013911) |
| **Length of Stay (LOS>7)** | | | | | | | |
| **Model** | **Task** | **AUROC** | **AUPRC** | **ACCURACY** | **PRECISION** | **RECALL** | **F1** |
| LR | LOS>7 | 0.683923 (0.020958) | 0.147404 (0.013944) | 0.918485 (0.001608) | 0.128000 (0.081953) | 0.011065 (0.007728) | 0.020369 (0.014275) |
| RF | LOS>7 | **0.753173 (0.018229)** | 0.198514 (0.018452) | **0.923411 (0.000000)** | 0.000000 (0.000000) | 0.000000 (0.000000) | 0.000000 (0.000000) |
| GRU-D | LOS>7 | 0.752725 (0.019067) | **0.203580 (0.021352)** | 0.920498 (0.001586) | **0.283465 (0.109889)** | **0.024896 (0.011499)** | **0.045772 (0.020523)** |
| TCN | LOS>7 | 0.749679 (0.018841) | 0.195810 (0.019228) | 0.920536 (0.001559) | 0.264706 (0.123853) | 0.019622 (0.010529) | 0.036536 (0.018776) |
| **In-ICU Mortality** | | | | | | | |
| **Model** | **Task** | **AUROC** | **AUPRC** | **ACCURACY** | **PRECISION** | **RECALL** | **F1** |
| LR | ICU-MORT | 0.841650 (0.015230) | 0.387721 (0.036147) | 0.931144 (0.003009) | 0.566219 (0.057757) | 0.215486 (0.033026) | 0.312169 (0.037366) |
| RF | ICU-MORT | 0.876558 (0.012744) | 0.466078 (0.036214) | 0.932309 (0.001644) | 0.837037 (0.086436) | 0.082542 (0.021561) | 0.150266 (0.034463) |
| GRU-D | ICU-MORT | **0.889758 (0.012482)** | **0.483509 (0.038389)** | **0.937553 (0.003620)** | **0.623057 (0.045253)** | **0.351351 (0.035960)** | **0.449323 (0.037248)** |
| TCN | ICU-MORT | 0.888429 (0.013003) | 0.479689 (0.037708) | 0.936049 (0.003799) | 0.599432 (0.047846) | 0.328149 (0.035317) | 0.424121 (0.039285) |
| **In-Hospital Mortality** | | | | | | | |
| **Model** | **Task** | **AUROC** | **AUPRC** | **ACCURACY** | **PRECISION** | **RECALL** | **F1** |
| LR | HOSP-MORT | 0.826968 (0.013152) | 0.435823 (0.031125) | 0.899364 (0.003955) | 0.584464 (0.045604) | 0.232695 (0.026700) | 0.332865 (0.032569) |
| RF | HOSP-MORT | 0.854973 (0.011533) | 0.497707 (0.029100) | 0.899110 (0.001907) | 0.870787 (0.072213) | 0.076092 (0.016083) | 0.139955 (0.028217) |
| GRU-D | HOSP-MORT | **0.874249 (0.010434)** | **0.535223 (0.027487)** | 0.909269 (0.004003) | 0.646739 (0.036893) | **0.350515 (0.028400)** | **0.454632 (0.030376)** |
| TCN | HOSP-MORT | 0.872179 (0.011617) | 0.527564 (0.031505**)** | **0.909654 (0.004297)** | **0.656379 (0.038152)** | 0.331773 (0.028882) | 0.440760 (0.031318) |

**Supplementary Appendix B – Brier Score Calibration Analysis**

Brier Scores represent the mean squared difference between the predicted probability and actual probability of outcome events, a method of measuring probability accuracy across a population. Here, we present Brier Score analysis for each model without rebalancing to show that the TCN has similar calibration performance to baselines.

Hold-Out Set Brier Score Plots:

| 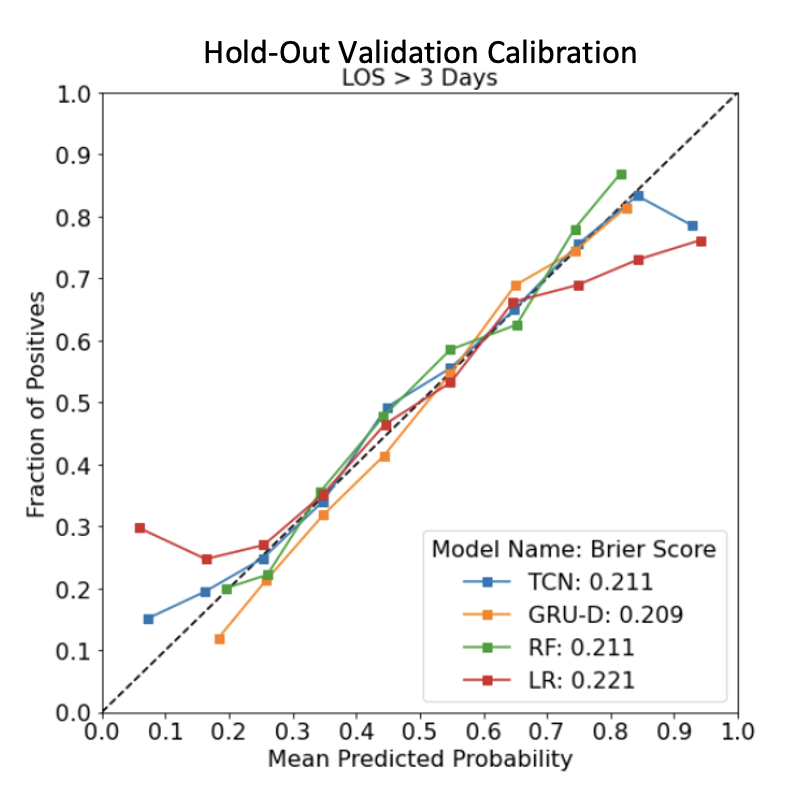 **Supplementary Figure 1:** LOS>3 hold-out calibration | 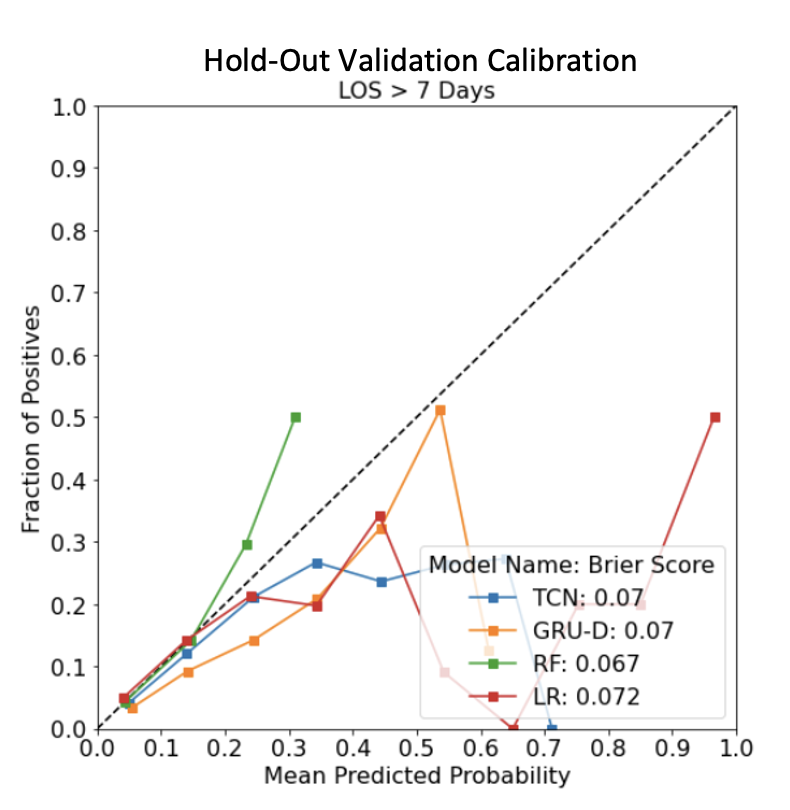 **Supplementary Figure 2:** LOS>7 hold-out calibration |
| --- | --- |
| 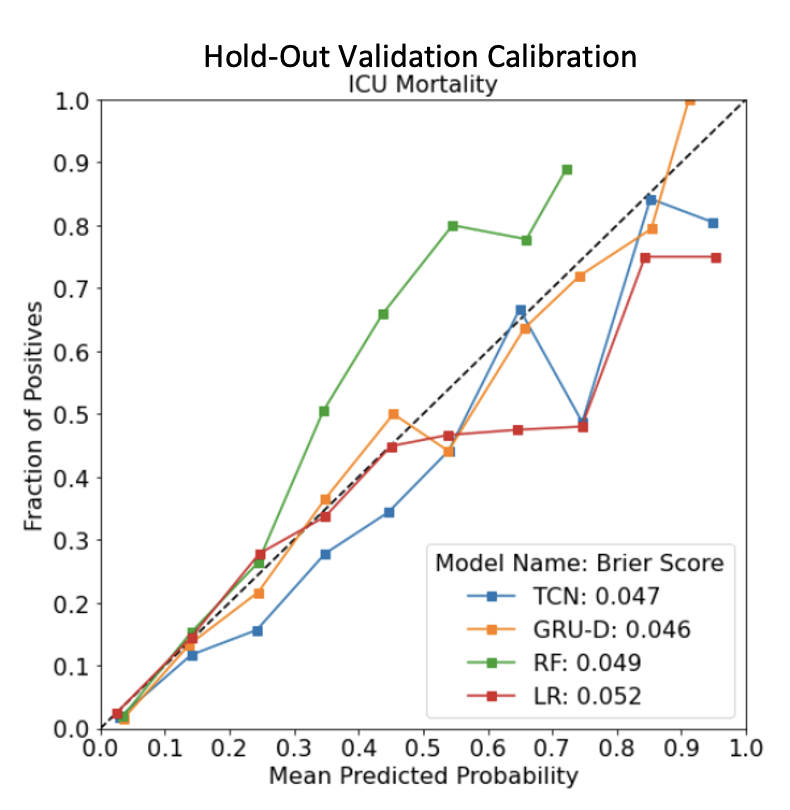 **Supplementary Figure 3:** ICU mortality hold-out calibration | 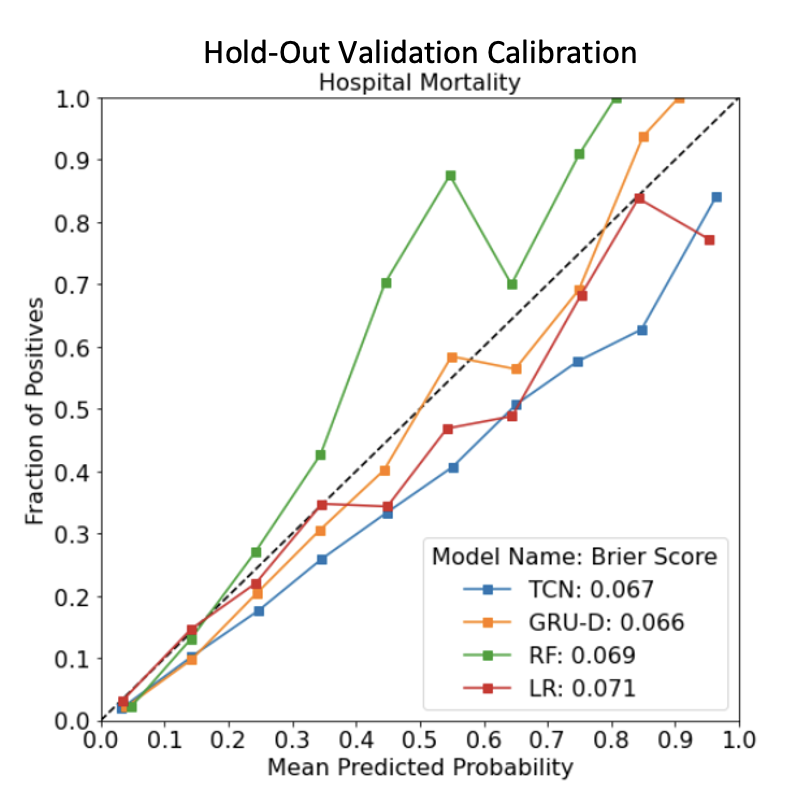 **Supplementary Figure 4:** hospital mortality hold-out calibration |

Cross-Validation Brier Score Plots:

| **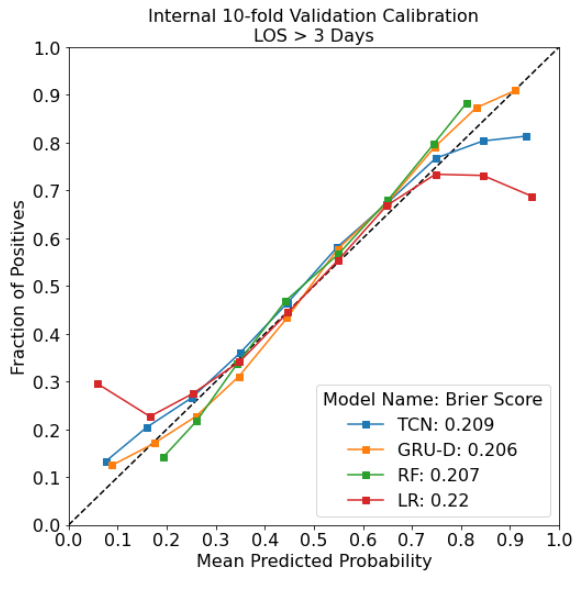** **Supplementary Figure 5:** LOS>3 cross-validation calibration | **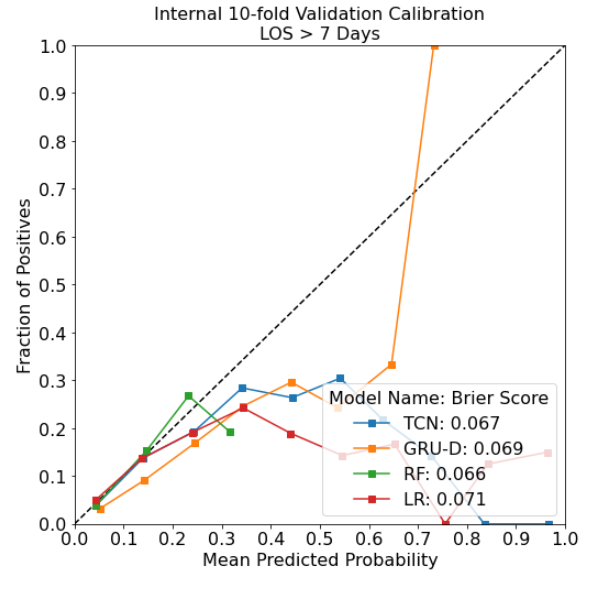** **Supplementary Figure 6:** LOS>7 cross-validation calibration |
| --- | --- |
| **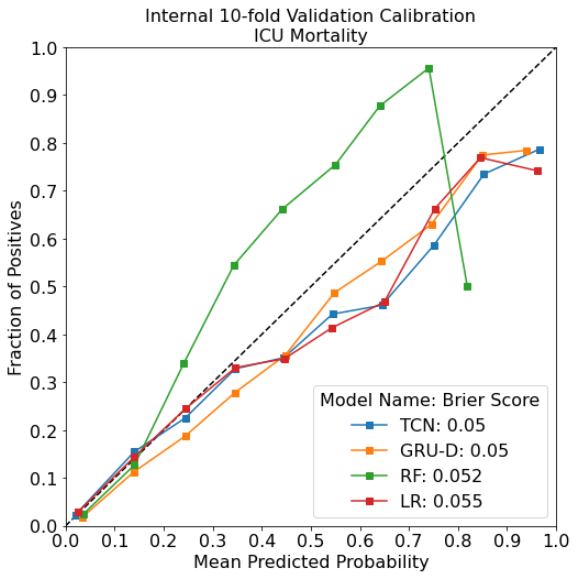** **Supplementary Figure 7:** ICU mortality cross-validation calibration | **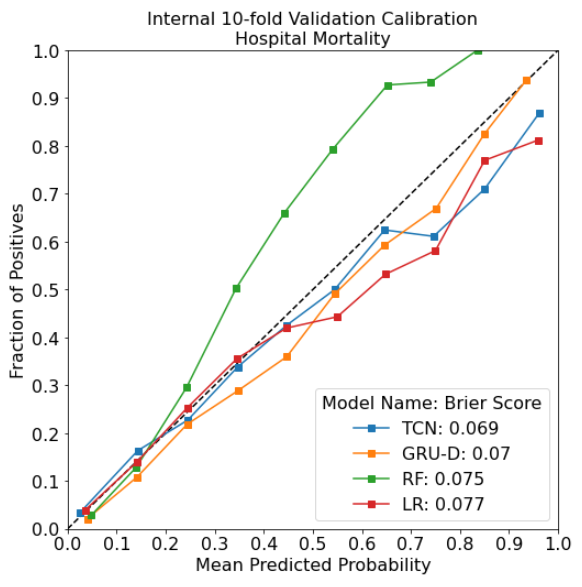** **Supplementary Figure 8:** hospital mortality cross-validation calibration |

**Supplementary Materials C –Rebalancing Methods and Complete Cross-Validation/Hold-out Results**

**Supplementary Table 2:** Complete Hold-out Rebalancing Results

| **LOS>3 days rebalancing** | | | | | | |
| --- | --- | --- | --- | --- | --- | --- |
| **Method** | **AUC** | **AUPRC** | **ACC** | **F1** | **Precision** | **Recall** |
| *SOTA From Any Model* | 0.721862 (0.020776) | 0.657199 (0.027243) | 0.680775 (0.017276) | 0.593656 (0.023937) | 0.660354 (0.027872) | 0.649518 (0.028350) |
| None | **0.716347 (0.020334)** | **0.649645 (0.027135)** | 0.670419 (0.016859) | 0.556235 (0.025789) | **0.660354 (0.027872)** | 0.480478 (0.029195) |
| Random Over-Sampling (1:1) | 0.712480 (0.020079) | 0.649091 (0.026961) | 0.667654 (0.017902) | 0.606316 (0.023193) | 0.617731 (0.022788) | 0.595315 (0.029550) |
| Random Under-Sampling (1:1) | 0.711865 (0.019982) | 0.643440 (0.026061) | **0.672788 (0.017803)** | 0.568826 (0.024282) | 0.656062 (0.027942) | 0.502067 (0.029331) |
| SMOTE (1:1) | 0.707812 (0.020707) | 0.644419 (0.026608) | 0.662322 (0.017787) | 0.613472 (0.022326) | 0.603916 (0.021209) | 0.623335 (0.029418) |
| Borderline-SMOTE (1:1) | 0.710096 (0.020487) | 0.643697 (0.025893) | 0.670221 (0.017966) | **0.628724 (0.021796)** | 0.609220 (0.021146) | **0.649518 (0.028350)** |
| SVM-SMOTE (1:1) | 0.702594 (0.020690) | 0.634746 (0.026055) | 0.669431 (0.017970) | 0.588698 (0.024179) | 0.632858 (0.023131) | 0.550299 (0.030307) |
| **LOS>7 days rebalancing** | | | | | | |
| **Method** | **AUC** | **AUPRC** | **ACC** | **F1** | **Precision** | **Recall** |
| *SOTA From Any Model* | 0.753061 (0.034916) | 0.224037 (0.048380) | 0.921209 (0.000387) | 0.098434 (0.054532) | 0.267148 (0.069424) | 0.781955 (0.057836) |
| None | 0.734888 (0.037653) | 0.187973 (0.035352) | **0.918049 (0.003176)** | 0.037123 (0.034763) | 0.250000 (0.218919) | 0.020050 (0.019406) |
| Random Over-Sampling (1:1) | 0.644341 (0.040998) | 0.139588 (0.027577) | 0.876777 (0.009493) | 0.174603 (0.052420) | 0.184874 (0.052437) | 0.165414 (0.051645) |
| Random Over-Sampling (1:2) | 0.663629 (0.040403) | 0.155957 (0.034890) | 0.885664 (0.009292) | 0.203576 (0.051879) | 0.225610 (0.058227) | 0.185464 (0.054216) |
| Random Over-Sampling (1:3) | 0.616650 (0.040552) | 0.132838 (0.031924) | 0.875000 (0.010200) | 0.157124 (0.049106) | 0.167614 (0.050690) | 0.147870 (0.047952) |
| Random Over-Sampling (1:4) | 0.696681 (0.038793) | 0.170196 (0.034494) | 0.879739 (0.009942) | 0.218228 (0.051374) | 0.223684 (0.054437) | 0.213033 (0.056601) |
| Random Over-Sampling (1:5) | 0.710851 (0.038837) | 0.177454 (0.033262) | 0.895735 (0.008481) | 0.187692 (0.055661) | 0.243028 (0.072675) | 0.152882 (0.051176) |
| Random Under-Sampling (1:1) | 0.734698 (0.036865) | **0.202788 (0.042753)** | 0.585900 (0.019263) | 0.229327 (0.016445) | 0.134367 (0.009907) | **0.781955 (0.057836)** |
| Random Under-Sampling (1:2) | 0.719753 (0.036176) | 0.192939 (0.041036) | 0.829384 (0.013997) | **0.267797 (0.043274)** | 0.202305 (0.032116) | 0.395990 (0.066942) |
| Random Under-Sampling (1:3) | 0.724463 (0.035940) | 0.183156 (0.034879) | 0.888626 (0.009983) | 0.225275 (0.056585) | 0.249240 (0.059130) | 0.205514 (0.054482) |
| Random Under-Sampling (1:4) | **0.746720 (0.033957)** | 0.198004 (0.040387) | 0.878949 (0.010874) | 0.258767 (0.053871) | 0.250000 (0.053598) | 0.268170 (0.060239) |
| Random Under-Sampling (1:5) | 0.736907 (0.036229) | 0.201786 (0.039724) | 0.895735 (0.009078) | 0.218935 (0.058707) | **0.267148 (0.069424)** | 0.185464 (0.054634) |
| SMOTE (1:1) | 0.648229 (0.040479) | 0.133342 (0.025542) | 0.876777 (0.009493) | 0.196396 (0.041551) | 0.153305 (0.032220) | 0.273183 (0.060071) |
| SMOTE (1:2) | 0.658666 (0.039480) | 0.142011 (0.028323) | 0.870853 (0.010673) | 0.186567 (0.049040) | 0.185185 (0.047466) | 0.187970 (0.054935) |
| SMOTE (1:3) | 0.629235 (0.040388) | 0.135533 (0.031073) | 0.873618 (0.010093) | 0.166667 (0.049265) | 0.173442 (0.049420) | 0.160401 (0.050382) |
| SMOTE (1:4) | 0.684157 (0.038553) | 0.159761 (0.033182) | 0.863152 (0.011424) | 0.213394 (0.051244) | 0.195021 (0.045495) | 0.235589 (0.059666) |
| SMOTE (1:5) | 0.698424 (0.039606) | 0.174396 (0.034432) | 0.898697 (0.008244) | 0.192126 (0.058971) | 0.258475 (0.075211) | 0.152882 (0.050822) |
| Borderline-SMOTE (1:1) | 0.638338 (0.041710) | 0.144492 (0.034745) | 0.891983 (0.008655) | 0.169954 (0.054507) | 0.215385 (0.065065) | 0.140351 (0.046511) |
| Borderline-SMOTE (1:2) | 0.660552 (0.039847) | 0.149304 (0.032832) | 0.901659 (0.006907) | 0.132404 (0.052288) | 0.217143 (0.082436) | 0.095238 (0.040718) |
| Borderline-SMOTE (1:3) | 0.650711 (0.042828) | 0.149694 (0.032213) | 0.892575 (0.007987) | 0.141956 (0.051919) | 0.191489 (0.066374) | 0.112782 (0.043410) |
| Borderline-SMOTE (1:4) | 0.671260 (0.040059) | 0.157152 (0.034431) | 0.872433 (0.010744) | 0.202469 (0.052413) | 0.199513 (0.049113) | 0.205514 (0.054632) |
| Borderline-SMOTE (1:5) | 0.684817 (0.039039) | 0.163562 (0.034104) | 0.899487 (0.007754) | 0.172358 (0.055968) | 0.245370 (0.078534) | 0.132832 (0.047156) |
| SVM-SMOTE (1:1) | 0.644718 (0.041707) | 0.138945 (0.027999) | 0.887046 (0.008695) | 0.143713 (0.050935) | 0.178439 (0.061703) | 0.120301 (0.046797) |
| SVM-SMOTE (1:2) | 0.672980 (0.038803) | 0.154145 (0.032522) | 0.891193 (0.008195) | 0.153610 (0.054318) | 0.198413 (0.064841) | 0.125313 (0.044742) |
| SVM-SMOTE (1:3) | 0.660793 (0.039875) | 0.142850 (0.026629) | 0.884281 (0.009252) | 0.155620 (0.051295) | 0.183051 (0.058568) | 0.135338 (0.046008) |
| SVM-SMOTE (1:4) | 0.665650 (0.039766) | 0.155813 (0.033185) | 0.880727 (0.009651) | 0.196809 (0.054422) | 0.209632 (0.056707) | 0.185464 (0.053408) |
| SVM-SMOTE (1:5) | 0.718498 (0.037787) | 0.185767 (0.037106) | 0.890008 (0.009397) | 0.198561 (0.054510) | 0.233108 (0.060556) | 0.172932 (0.051375) |
| **In-Hospital Mortality (hosp_mort) rebalancing** | | | | | | |
| **Method** | **AUC** | **AUPRC** | **ACC** | **F1** | **Precision** | **Recall** |
| *SOTA From Any Model* | 0.894402 (0.022971) | 0.507862 (0.071698) | 0.942536 (0.006484) | 0.466055 (0.073581) | 0.644670 (0.087217) | 0.810345 (0.058447) |
| None | **0.892135 (0.025004)** | 0.507862 (0.071698) | **0.942536 (0.006484)** | 0.466055 (0.073581) | **0.644670 (0.087217)** | 0.364943 (0.071146) |
| Random Over-Sampling (1:1) | 0.843564 (0.024801) | 0.442745 (0.059194) | 0.850118 (0.013684) | 0.445581 (0.038639) | 0.444767 (0.066014) | 0.439655 (0.074621) |
| Random Over-Sampling (1:2) | 0.843053 (0.026871) | 0.470630 (0.059014) | 0.891983 (0.011029) | 0.474544 (0.052232) | 0.485149 (0.072292) | 0.422414 (0.074527) |
| Random Over-Sampling (1:3) | 0.857310 (0.024540) | 0.511827 (0.061836) | 0.910545 (0.009191) | 0.491582 (0.057283) | 0.409953 (0.055458) | 0.497126 (0.074994) |
| Random Over-Sampling (1:4) | 0.862980 (0.021624) | 0.505109 (0.060467) | 0.910150 (0.008268) | 0.433375 (0.057655) | 0.419598 (0.059746) | 0.479885 (0.072812) |
| Random Over-Sampling (1:5) | 0.863025 (0.024306) | 0.509293 (0.057894) | 0.897907 (0.010968) | **0.492640 (0.051405)** | 0.341935 (0.039032) | 0.609195 (0.073706) |
| Random Under-Sampling (1:1) | 0.866100 (0.022471) | 0.479734 (0.060957) | 0.734202 (0.016979) | 0.383135 (0.022963) | 0.222749 (0.018533) | **0.810345 (0.058447)** |
| Random Under-Sampling (1:2) | 0.869164 (0.023321) | 0.488484 (0.059038) | 0.866706 (0.012881) | 0.492099 (0.039823) | 0.354890 (0.039850) | 0.646552 (0.069709) |
| Random Under-Sampling (1:3) | 0.870307 (0.021453) | 0.504261 (0.060562) | 0.877370 (0.012252) | 0.485501 (0.044218) | 0.377049 (0.044854) | 0.594828 (0.073677) |
| Random Under-Sampling (1:4) | 0.872939 (0.021837) | 0.521897 (0.058278) | 0.908175 (0.009690) | 0.489572 (0.055296) | 0.387097 (0.044466) | 0.586207 (0.073709) |
| Random Under-Sampling (1:5) | 0.875360 (0.021592) | 0.516751 (0.061405) | 0.911532 (0.008994) | 0.489749 (0.056697) | 0.439803 (0.057235) | 0.514368 (0.073823) |
| SMOTE (1:1) | 0.838810 (0.027404) | 0.465919 (0.056245) | 0.877567 (0.012030) | 0.450355 (0.047929) | 0.459155 (0.062771) | 0.468391 (0.070145) |
| SMOTE (1:2) | 0.845501 (0.026387) | 0.483223 (0.060407) | 0.890995 (0.010639) | 0.466151 (0.051777) | 0.443590 (0.060867) | 0.497126 (0.074500) |
| SMOTE (1:3) | 0.851026 (0.023761) | 0.482724 (0.060223) | 0.905608 (0.009251) | 0.462921 (0.054536) | 0.456869 (0.068845) | 0.410920 (0.071006) |
| SMOTE (1:4) | 0.858495 (0.024234) | 0.506416 (0.058385) | 0.907978 (0.009640) | 0.486784 (0.055374) | 0.421951 (0.054566) | 0.497126 (0.072527) |
| SMOTE (1:5) | 0.859990 (0.023451) | 0.503262 (0.059635) | 0.909558 (0.008583) | 0.442822 (0.059059) | 0.501754 (0.069804) | 0.410920 (0.071673) |
| Borderline-SMOTE (1:1) | 0.845612 (0.025885) | 0.463935 (0.057957) | 0.897117 (0.009965) | 0.460104 (0.053384) | 0.446154 (0.065090) | 0.416667 (0.070384) |
| Borderline-SMOTE (1:2) | 0.853850 (0.024925) | 0.489457 (0.059739) | 0.899092 (0.010080) | 0.471562 (0.055022) | 0.510417 (0.071167) | 0.422414 (0.074742) |
| Borderline-SMOTE (1:3) | 0.859677 (0.023840) | 0.505686 (0.058034) | 0.906596 (0.009210) | 0.486428 (0.054189) | 0.537102 (0.071069) | 0.436782 (0.075092) |
| Borderline-SMOTE (1:4) | 0.846919 (0.024800) | 0.485488 (0.057628) | 0.901461 (0.010017) | 0.473073 (0.053042) | 0.434783 (0.061546) | 0.459770 (0.074652) |
| Borderline-SMOTE (1:5) | 0.873931 (0.022878) | 0.524914 (0.056558) | 0.904818 (0.009555) | 0.485043 (0.055632) | 0.402464 (0.047580) | 0.563218 (0.071275) |
| SVM-SMOTE (1:1) | 0.851021 (0.023915) | 0.478716 (0.059673) | 0.888823 (0.011171) | 0.461244 (0.051011) | 0.421836 (0.055945) | 0.488506 (0.072723) |
| SVM-SMOTE (1:2) | 0.858640 (0.024563) | 0.505769 (0.057135) | 0.903633 (0.009546) | 0.458980 (0.054756) | 0.525952 (0.075146) | 0.436782 (0.071302) |
| SVM-SMOTE (1:3) | 0.865055 (0.023831) | 0.508569 (0.056289) | 0.904621 (0.010073) | 0.486716 (0.052813) | 0.584906 (0.086101) | 0.356322 (0.069876) |
| SVM-SMOTE (1:4) | 0.866467 (0.023242) | 0.512835 (0.060092) | 0.910742 (0.008793) | 0.459330 (0.059150) | 0.447090 (0.061771) | 0.485632 (0.074479) |
| SVM-SMOTE (1:5) | 0.874008 (0.022974) | **0.530713 (0.061173)** | 0.906793 (0.009702) | 0.490281 (0.055903) | 0.431507 (0.056011) | 0.543103 (0.074228) |
| **In-ICU Mortality (icu_mort) rebalancing** | | | | | | |
| **Method** | **AUC** | **AUPRC** | **ACC** | **F1** | **Precision** | **Recall** |
| *SOTA From Any Model* | 0.877008 (0.021963) | 0.530339 (0.058580) | 0.916139 (0.007674) | 0.472123 (0.060955) | 0.587021 (0.067176) | 0.829365 (0.046421) |
| None | 0.877008 (0.021963) | **0.530339 (0.058580)** | 0.912125 (0.008698) | 0.472123 (0.060955) | **0.587021 (0.067176)** | 0.394841 (0.062229) |
| Random Over-Sampling (1:1) | 0.848410 (0.031665) | 0.420873 (0.074474) | 0.923776 (0.008842) | 0.442197 (0.064508) | 0.352601 (0.033788) | 0.605159 (0.060602) |
| Random Over-Sampling (1:2) | 0.871134 (0.026891) | 0.438669 (0.068913) | 0.929502 (0.008421) | 0.451613 (0.062535) | 0.459963 (0.052126) | 0.490079 (0.061851) |
| Random Over-Sampling (1:3) | 0.877281 (0.026131) | 0.454945 (0.072677) | 0.916272 (0.009634) | 0.449351 (0.056184) | 0.565891 (0.062481) | 0.434524 (0.060879) |
| Random Over-Sampling (1:4) | 0.883421 (0.024665) | 0.455104 (0.068087) | 0.918641 (0.009661) | 0.447721 (0.058395) | 0.581940 (0.073162) | 0.345238 (0.059360) |
| Random Over-Sampling (1:5) | 0.883927 (0.025397) | 0.471708 (0.072878) | 0.892575 (0.011467) | 0.438017 (0.045629) | 0.487379 (0.053681) | 0.498016 (0.060970) |
| Random Under-Sampling (1:1) | 0.887818 (0.022945) | 0.450846 (0.071461) | 0.792654 (0.016020) | 0.349442 (0.026719) | 0.249106 (0.016384) | **0.829365 (0.046421)** |
| Random Under-Sampling (1:2) | 0.891334 (0.024740) | 0.471179 (0.070224) | 0.894945 (0.011771) | 0.458248 (0.045768) | 0.396364 (0.034827) | 0.648810 (0.058597) |
| Random Under-Sampling (1:3) | 0.890270 (0.023515) | 0.455455 (0.071414) | 0.904621 (0.011605) | 0.461538 (0.050499) | 0.416785 (0.040804) | 0.581349 (0.059941) |
| Random Under-Sampling (1:4) | 0.892526 (0.022171) | 0.484892 (0.069617) | 0.907780 (0.011034) | 0.466286 (0.053112) | 0.547912 (0.061813) | 0.442460 (0.060963) |
| Random Under-Sampling (1:5) | **0.902723 (0.021827)** | 0.514730 (0.068023) | 0.921603 (0.009927) | 0.474172 (0.058112) | 0.574866 (0.065730) | 0.426587 (0.058274) |
| SMOTE (1:1) | 0.858142 (0.029661) | 0.440614 (0.069794) | 0.925553 (0.009278) | 0.463727 (0.062813) | 0.407051 (00.044800 | 0.503968 (0.060528) |
| SMOTE (1:2) | 0.860390 (0.029299) | 0.419838 (0.072791) | 0.922591 (0.009448) | 0.468835 (0.058322) | 0.454717 (0.051146) | 0.478175 (0.062672) |
| SMOTE (1:3) | 0.864511 (0.027199) | 0.424790 (0.070518) | 0.925948 (0.008495) | 0.432678 (0.064882) | 0.533679 (0.062252) | 0.408730 (0.060020) |
| SMOTE (1:4) | 0.865030 (0.027890) | 0.440317 (0.071883) | 0.918641 (0.009788) | 0.456464 (0.058018) | 0.547030 (0.060501) | 0.438492 (0.061764) |
| SMOTE (1:5) | 0.862077 (0.028890) | 0.442452 (0.075762) | 0.931477 (0.008126) | 0.451817 (0.067311) | 0.572327 (0.070845) | 0.361111 (0.057674) |
| Borderline-SMOTE (1:1) | 0.868829 (0.028048) | 0.449265 (0.071883) | 0.924368 (0.008707) | 0.430906 (0.063606) | 0.481562 (0.056282) | 0.440476 (0.060468) |
| Borderline-SMOTE (1:2) | 0.865902 (0.027587) | 0.444977 (0.075961) | 0.932464 (0.008501) | 0.462264 (0.066849) | 0.492441 (0.056675) | 0.452381 (0.062526) |
| Borderline-SMOTE (1:3) | 0.869361 (0.027311) | 0.452493 (0.074711) | 0.935427 (0.007763) | **0.481775 (0.067925)** | 0.537170 (0.061324) | 0.444444 (0.059257) |
| Borderline-SMOTE (1:4) | 0.873128 (0.026763) | 0.457140 (0.070803) | 0.921801 (0.009558) | 0.446927 (0.059283) | 0.505643 (0.057290) | 0.444444 (0.061845) |
| Borderline-SMOTE (1:5) | 0.878663 (0.028312) | 0.483810 (0.069682) | 0.912520 (0.010175) | 0.469461 (0.054122) | 0.525463 (0.059406) | 0.450397 (0.067391) |
| SVM-SMOTE (1:1) | 0.875597 (0.025065) | 0.446416 (0.073784) | 0.918839 (0.009348) | 0.452730 (0.058600) | 0.445471 (00.052929 | 0.478175 (0.062088) |
| SVM-SMOTE (1:2) | 0.875133 (0.026348) | 0.454480 (0.073053) | 0.934242 (0.007771) | 0.477237 (0.066690) | 0.520101 (0.061036) | 0.410714 (0.060917) |
| SVM-SMOTE (1:3) | 0.880753 (0.024298) | 0.466517 (0.074857) | **0.938389 (0.006973)** | 0.442857 (0.070795) | 0.524027 (0.057237) | 0.454365 (0.063820) |
| SVM-SMOTE (1:4) | 0.878692 (0.025645) | 0.468890 (0.071027) | 0.923381 (0.009330) | 0.465565 (0.057926) | 0.578313 (0.066990) | 0.380952 (0.057972) |
| SVM-SMOTE (1:5) | 0.890626 (0.025526) | 0.492410 (0.073611) | 0.919431 (0.009937) | 0.480916 (0.057240) | 0.537915 (0.059445) | 0.450397 (0.060028) |

**Supplementary Table 3:** Complete Cross-Validation Rebalancing Results

| **LOS>3 days rebalancing** | | | | | | |
| --- | --- | --- | --- | --- | --- | --- |
| **Method** | **AUC** | **AUPRC** | **ACC** | **F1** | **Precision** | **Recall** |
| *SOTA From Any Model* | **0.729915 (0.010146)** | **0.681426 (0.013544)** | **0.690837 (0.008618)** | 0.589320 (0.012894) | 0.684113 (0.015018) | 0.617967 (0.015747) |
| None | 0.722935 (0.010514) | 0.660374 (0.014704) | 0.680636 (0.008822) | 0.563829 (0.013911) | **0.684113 (0.015018)** | 0.479518 (0.015951) |
| Random Over-Sampling (1:1) | 0.710233 (0.010442) | 0.653141 (0.014508) | 0.668248 (0.009693) | 0.604063 (0.012413) | 0.621148 (0.012331) | 0.587892 (0.015705) |
| Random Under-Sampling (1:1) | 0.708574 (0.010743) | 0.647340 (0.014610) | 0.667690 (0.009541) | 0.607216 (0.012116) | 0.618101 (0.011787) | 0.596707 (0.014992) |
| SMOTE (1:1) | 0.706976 (0.011007) | 0.650961 (0.013924) | 0.663281 (0.009626) | **0.608233 (0.011906)** | 0.609261 (0.011766) | 0.607208 (0.014812) |
| Borderline-SMOTE (1:1) | 0.703028 (0.010730) | 0.649288 (0.014498) | 0.655525 (0.009503) | 0.606991 (0.011793) | 0.596397 (0.011424) | **0.617967 (0.015747)** |
| SVM-SMOTE (1:1) | 0.705833 (0.010956) | 0.647749 (0.014017) | 0.664788 (0.009665) | 0.596602 (0.012193) | 0.618922 (0.012331) | 0.575836 (0.014725) |
| **LOS>7 days rebalancing** | | | | | | |
| **Method** | **AUC** | **AUPRC** | **ACC** | **F1** | **Precision** | **Recall** |
| *SOTA From Any Model* | **0.753173 (0.018229)** | **0.203580 (0.021352)** | **0.923411 (0.000000)** | 0.045772 (0.020523) | 0.264706 (0.123853) | 0.674419 (0.034043) |
| None | 0.749679 (0.018841) | 0.195810 (0.019228) | 0.920536 (0.001559) | 0.036536 (0.018776) | **0.264706 (0.123853)** | 0.019622 (0.010529) |
| Random Over-Sampling (1:1) | 0.653650 (0.021093) | 0.134839 (0.014359) | 0.849609 (0.006399) | 0.236614 (0.011376) | 0.160227 (0.020688) | 0.226017 (0.031483) |
| Random Over-Sampling (1:2) | 0.653873 (0.021461) | 0.134092 (0.013230) | 0.882143 (0.005116) | **0.261395 (0.023172)** | 0.180000 (0.029907) | 0.150436 (0.026620) |
| Random Over-Sampling (1:3) | 0.673089 (0.021034) | 0.146716 (0.015621) | 0.886942 (0.004825) | 0.231549 (0.029138) | 0.199074 (0.031146) | 0.156250 (0.026595) |
| Random Over-Sampling (1:4) | 0.683990 (0.021401) | 0.154955 (0.016977) | 0.888951 (0.004994) | 0.209349 (0.030145) | 0.218349 (0.031619) | 0.172965 (0.028176) |
| Random Over-Sampling (1:5) | 0.700958 (0.020787) | 0.160209 (0.016393) | 0.896540 (0.004334) | 0.152099 (0.029948) | 0.220141 (0.037126) | 0.136628 (0.025849) |
| Random Under-Sampling (1:1) | 0.722699 (0.019796) | 0.168695 (0.015896) | 0.665848 (0.009735) | 0.187519 (0.023517) | 0.143476 (0.007279) | **0.674419 (0.034043)** |
| Random Under-Sampling (1:2) | 0.724598 (0.019992) | 0.176491 (0.017414) | 0.836328 (0.007006) | 0.163895 (0.027706) | 0.200000 (0.017148) | 0.377180 (0.036427) |
| Random Under-Sampling (1:3) | 0.726035 (0.018794) | 0.174989 (0.016469) | 0.877400 (0.005750) | 0.175081 (0.028888) | 0.223196 (0.026975) | 0.240552 (0.032218) |
| Random Under-Sampling (1:4) | 0.734240 (0.019517) | 0.179457 (0.017763) | 0.889565 (0.004916) | 0.193025 (0.028565) | 0.232476 (0.033356) | 0.190407 (0.029614) |
| Random Under-Sampling (1:5) | 0.728848 (0.018851) | 0.178925 (0.018564) | 0.904185 (0.003658) | 0.168610 (0.030089) | 0.237288 (0.045306) | 0.111919 (0.023467) |
| SMOTE (1:1) | 0.642974 (0.022038) | 0.125903 (0.013031) | 0.855078 (0.006206) | 0.174245 (0.024403) | 0.154890 (0.021381) | 0.199128 (0.030262) |
| SMOTE (1:2) | 0.667602 (0.021112) | 0.140902 (0.014405) | 0.876786 (0.005132) | 0.186441 (0.027698) | 0.189088 (0.027495) | 0.183866 (0.029943) |
| SMOTE (1:3) | 0.654421 (0.021881) | 0.139183 (0.015540) | 0.892801 (0.004440) | 0.160035 (0.028230) | 0.200878 (0.035077) | 0.132994 (0.025792) |
| SMOTE (1:4) | 0.667217 (0.021171) | 0.144800 (0.015685) | 0.876618 (0.005272) | 0.192771 (0.028649) | 0.193690 (0.027222) | 0.191860 (0.028881) |
| SMOTE (1:5) | 0.692417 (0.020109) | 0.158518 (0.016482) | 0.897824 (0.004328) | 0.161246 (0.028573) | 0.218092 (0.037225) | 0.127907 (0.024391) |
| Borderline-SMOTE (1:1) | 0.647513 (0.021785) | 0.130234 (0.013595) | 0.875670 (0.005434) | 0.168036 (0.026461) | 0.172811 (0.026586) | 0.163517 (0.026385) |
| Borderline-SMOTE (1:2) | 0.652823 (0.022640) | 0.134288 (0.014195) | 0.885379 (0.004905) | 0.156122 (0.026588) | 0.179584 (0.030745) | 0.138081 (0.026219) |
| Borderline-SMOTE (1:3) | 0.667991 (0.021772) | 0.147236 (0.015994) | 0.900558 (0.003964) | 0.148997 (0.028324) | 0.217270 (0.042095) | 0.113372 (0.023511) |
| Borderline-SMOTE (1:4) | 0.676481 (0.021473) | 0.150564 (0.016260) | 0.892913 (0.004605) | 0.175333 (0.029557) | 0.214511 (0.035438) | 0.148256 (0.025843) |
| Borderline-SMOTE (1:5) | 0.685032 (0.020167) | 0.156367 (0.017006) | 0.894810 (0.004489) | 0.184336 (0.029382) | 0.227807 (0.035393) | 0.154797 (0.027159) |
| SVM-SMOTE (1:1) | 0.655777 (0.021468) | 0.131408 (0.012656) | 0.877344 (0.005261) | 0.150696 (0.027026) | 0.160891 (0.027881) | 0.141715 (0.026450) |
| SVM-SMOTE (1:2) | 0.657380 (0.021420) | 0.136522 (0.014066) | 0.890234 (0.004717) | 0.160478 (0.027828) | 0.194416 (0.032610) | 0.136628 (0.025822) |
| SVM-SMOTE (1:3) | 0.671819 (0.021456) | 0.146051 (0.015784) | 0.885603 (0.005006) | 0.174053 (0.027212) | 0.195298 (0.029304) | 0.156977 (0.026875) |
| SVM-SMOTE (1:4) | 0.691387 (0.020752) | 0.156504 (0.016280) | 0.901618 (0.004206) | 0.164059 (0.030265) | 0.236016 (0.039968) | 0.125727 (0.024560) |
| SVM-SMOTE (1:5) | 0.701166 (0.019705) | 0.162987 (0.016383) | 0.906194 (0.003558) | 0.133058 (0.028303) | 0.229130 (0.046361) | 0.093750 (0.021832) |
| **In-Hospital Mortality (hosp_mort) rebalancing** | | | | | | |
| **Method** | **AUC** | **AUPRC** | **ACC** | **F1** | **Precision** | **Recall** |
| *SOTA From Any Model* | **0.889758 (0.012482)** | 0.483509 (0.038389) | **0.937553 (0.003620)** | 0.449323 (0.037248) | 0.656379 (0.038152) | 0.761310 (0.026369) |
| None | 0.888429 (0.013003) | 0.479689 (0.037708) | 0.936049 (0.003799) | 0.424121 (0.039285) | **0.656379 (0.038152)** | 0.331773 (0.028882) |
| Random Over-Sampling (1:1) | 0.835543 (0.013482) | 0.465662 (0.031135) | 0.862891 (0.006815) | 0.461302 (0.022956) | 0.398787 (0.021950) | 0.547062 (0.031698) |
| Random Over-Sampling (1:2) | 0.845879 (0.013614) | 0.493414 (0.030453) | 0.882645 (0.005815) | 0.483419 (0.024705) | 0.458101 (0.025040) | 0.511700 (0.032461) |
| Random Over-Sampling (1:3) | 0.854734 (0.013571) | 0.499748 (0.030542) | 0.892969 (0.006068) | 0.490706 (0.027093) | 0.501356 (0.027481) | 0.480499 (0.030821) |
| Random Over-Sampling (1:4) | 0.854918 (0.012987) | 0.513360 (0.030154) | 0.897210 (0.005750) | 0.496996 (0.027002) | 0.523289 (0.028470) | 0.473219 (0.032159) |
| Random Over-Sampling (1:5) | 0.854935 (0.012340) | 0.513082 (0.030208) | 0.898772 (0.005215) | 0.472061 (0.029122) | 0.536021 (0.030098) | 0.421737 (0.031033) |
| Random Under-Sampling (1:1) | 0.858048 (0.011562) | 0.468138 (0.030917) | 0.795368 (0.007850) | 0.443973 (0.015548) | 0.313356 (0.012371) | **0.761310 (0.026369)** |
| Random Under-Sampling (1:2) | 0.861980 (0.011617) | 0.495304 (0.030647) | 0.864342 (0.006596) | 0.485285 (0.022287) | 0.409286 (0.020051) | 0.595944 (0.029735) |
| Random Under-Sampling (1:3) | 0.862827 (0.011452) | 0.499292 (0.031009) | 0.875167 (0.006491) | 0.489852 (0.023929) | 0.436231 (0.022460) | 0.558502 (0.031223) |
| Random Under-Sampling (1:4) | 0.867402 (0.011861) | 0.516212 (0.030340) | 0.892801 (0.005881) | **0.504002 (0.025686)** | 0.500513 (0.026400) | 0.507540 (0.032978) |
| Random Under-Sampling (1:5) | 0.861515 (0.012145) | 0.509772 (0.031067) | 0.900502 (0.005114) | 0.479112 (0.028194) | 0.546667 (0.032675) | 0.426417 (0.031041) |
| SMOTE (1:1) | 0.828976 (0.013610) | 0.460614 (0.031532) | 0.863504 (0.006718) | 0.460282 (0.022571) | 0.399770 (0.020862) | 0.542382 (0.032287) |
| SMOTE (1:2) | 0.832535 (0.013218) | 0.462587 (0.030437) | 0.869364 (0.006548) | 0.449306 (0.024345) | 0.410223 (0.023130) | 0.496620 (0.032092) |
| SMOTE (1:3) | 0.847092 (0.013217) | 0.495400 (0.030449) | 0.883482 (0.006040) | 0.475377 (0.026580) | 0.459893 (0.026061) | 0.491940 (0.031204) |
| SMOTE (1:4) | 0.846725 (0.012890) | 0.496447 (0.031604) | 0.891685 (0.005659) | 0.488268 (0.026379) | 0.495187 (0.028115) | 0.481539 (0.031537) |
| SMOTE (1:5) | 0.860658 (0.011780) | 0.520528 (0.030636) | 0.898884 (0.005444) | 0.488136 (0.028424) | 0.534323 (0.030192) | 0.449298 (0.032844) |
| Borderline-SMOTE (1:1) | 0.832291 (0.013792) | 0.468831 (0.031572) | 0.875446 (0.006511) | 0.465773 (0.024430) | 0.431486 (0.024132) | 0.505980 (0.030596) |
| Borderline-SMOTE (1:2) | 0.842836 (0.012796) | 0.494591 (0.030950) | 0.887054 (0.005941) | 0.477003 (0.025653) | 0.474063 (0.026240) | 0.479979 (0.031038) |
| Borderline-SMOTE (1:3) | 0.847932 (0.012902) | 0.501815 (0.030033) | 0.890067 (0.005467) | 0.490166 (0.026455) | 0.487893 (0.027044) | 0.492460 (0.031336) |
| Borderline-SMOTE (1:4) | 0.858424 (0.012409) | 0.512150 (0.031131) | 0.895089 (0.005610) | 0.496518 (0.027055) | 0.511872 (0.027146) | 0.482059 (0.032229) |
| Borderline-SMOTE (1:5) | 0.857807 (0.012878) | **0.522999 (0.030591)** | 0.900558 (0.005254) | 0.487047 (0.027561) | 0.545455 (0.030726) | 0.439938 (0.032099) |
| SVM-SMOTE (1:1) | 0.834798 (0.013883) | 0.472894 (0.032008) | 0.875837 (0.006116) | 0.475360 (0.024472) | 0.434858 (0.023400) | 0.524181 (00.030773 |
| SVM-SMOTE (1:2) | 0.844353 (0.013544) | 0.497424 (0.031380) | 0.892857 (0.005517) | 0.479675 (0.027900) | 0.500849 (0.028291) | 0.460218 (0.031533) |
| SVM-SMOTE (1:3) | 0.857345 (0.012712) | 0.516200 (0.030400) | 0.897545 (0.005453) | 0.495050 (0.027008) | 0.525394 (0.029575) | 0.468019 (0.031646) |
| SVM-SMOTE (1:4) | 0.858573 (0.012340) | 0.516967 (0.030508) | 0.901842 (0.005248) | 0.492059 (0.027381) | 0.553247 (0.030903) | 0.443058 (0.031017) |
| SVM-SMOTE (1:5) | 0.861342 (0.012424) | 0.519882 (0.030524) | 0.904297 (0.005059) | 0.481717 (0.029360) | 0.575036 (0.032304) | 0.414457 (0.030548) |
| **In-ICU Mortality (icu_mort) rebalancing** | | | | | | |
| **Method** | **AUC** | **AUPRC** | **ACC** | **F1** | **Precision** | **Recall** |
| *SOTA From Any Model* | 0.881579 (0.013740) | **0.527564 (0.031505)** | **0.909654 (0.004297)** | 0.454632 (0.030376) | 0.599432 (0.047846) | 0.803266 (0.031252) |
| None | 0.872179 (0.011617) | **0.527564 (0.031505)** | **0.909654 (0.004297)** | 0.440760 (0.031318) | **0.599432 (0.047846)** | 0.328149 (0.035317) |
| Random Over-Sampling (1:1) | 0.858445 (0.014995) | 0.432872 (0.038611) | 0.913058 (0.005308) | 0.448300 (0.030336) | 0.411573 (0.028196) | 0.492224 (0.038883) |
| Random Over-Sampling (1:2) | 0.864511 (0.015203) | 0.430786 (0.037561) | 0.913895 (0.005171) | 0.447943 (0.029817) | 0.414844 (0.028903) | 0.486781 (0.039082) |
| Random Over-Sampling (1:3) | 0.863455 (0.015700) | 0.454233 (0.039038) | 0.923382 (0.004814) | 0.462201 (0.032359) | 0.465667 (0.033470) | 0.458787 (0.038015) |
| Random Over-Sampling (1:4) | 0.863879 (0.015438) | 0.461384 (0.038845) | 0.925167 (0.004547) | 0.461229 (0.032642) | 0.477140 (0.035159) | 0.446345 (0.038077) |
| Random Over-Sampling (1:5) | 0.862566 (0.015289) | 0.442238 (0.039622) | 0.927511 (0.004355) | 0.457620 (0.033422) | 0.494139 (0.036932) | 0.426128 (0.037996) |
| Random Under-Sampling (1:1) | 0.874258 (0.013366) | 0.418994 (0.038301) | 0.786440 (0.008232) | 0.350585 (0.013459) | 0.224224 (0.009325) | **0.803266 (0.031252)** |
| Random Under-Sampling (1:2) | 0.879420 (0.013141) | 0.443433 (0.038904) | 0.877232 (0.006865) | 0.426486 (0.023357) | 0.320784 (0.018942) | 0.636081 (0.036713) |
| Random Under-Sampling (1:3) | 0.879442 (0.013898) | 0.454439 (0.039017) | 0.909263 (0.005665) | 0.460159 (0.028451) | 0.401506 (0.026189) | 0.538880 (0.037391) |
| Random Under-Sampling (1:4) | 0.879307 (0.013357) | 0.459220 (0.039012) | 0.916908 (0.005188) | 0.469540 (0.030606) | 0.433268 (0.028989) | 0.512442 (0.038173) |
| Random Under-Sampling (1:5) | **0.881579 (0.013740)** | 0.463848 (0.038842) | 0.925000 (0.004833) | **0.481881 (0.031657)** | 0.477829 (0.032939) | 0.486003 (0.039477) |
| SMOTE (1:1) | 0.846712 (0.016441) | 0.418490 (0.036343) | 0.896875 (0.005849) | 0.433129 (0.026534) | 0.357649 (0.024087) | 0.548989 (0.035909) |
| SMOTE (1:2) | 0.854788 (0.015726) | 0.424151 (0.038213) | 0.912388 (0.005389) | 0.438885 (0.030811) | 0.406085 (0.029441) | 0.477449 (0.038749) |
| SMOTE (1:3) | 0.858861 (0.014570) | 0.442265 (0.039635) | 0.921819 (0.004897) | 0.454652 (0.030941) | 0.455183 (0.033654) | 0.454121 (0.040738) |
| SMOTE (1:4) | 0.860241 (0.015297) | 0.451008 (0.037166) | 0.917076 (0.005168) | 0.450037 (0.031369) | 0.429379 (0.030463) | 0.472784 (0.038963) |
| SMOTE (1:5) | 0.861466 (0.014552) | 0.447785 (0.038437) | 0.920368 (0.004817) | 0.449247 (0.032177) | 0.445977 (0.031507) | 0.452566 (0.037437) |
| Borderline-SMOTE (1:1) | 0.852401 (0.015829) | 0.432238 (0.038651) | 0.916016 (0.005103) | 0.448920 (0.029956) | 0.424221 (0.029763) | 0.476672 (0.037914) |
| Borderline-SMOTE (1:2) | 0.859952 (0.014954) | 0.446643 (0.037794) | 0.921987 (0.005033) | 0.466005 (0.031618) | 0.457958 (0.031220) | 0.474339 (0.038295) |
| Borderline-SMOTE (1:3) | 0.868156 (0.015075) | 0.472835 (0.038865) | 0.928237 (0.004543) | 0.472951 (0.034110) | 0.500000 (0.035240) | 0.448678 (0.036911) |
| Borderline-SMOTE (1:4) | 0.872016 (0.014832) | 0.473365 (0.037011) | 0.925893 (0.004493) | 0.466667 (0.031445) | 0.482558 (0.035816) | 0.451788 (0.038640) |
| Borderline-SMOTE (1:5) | 0.865559 (0.015337) | 0.467998 (0.038585) | 0.925781 (0.004933) | 0.466720 (0.031923) | 0.481788 (0.033713) | 0.452566 (0.039486) |
| SVM-SMOTE (1:1) | 0.854085 (0.016018) | 0.437147 (0.037829) | 0.911663 (0.005324) | 0.447855 (0.030323) | 0.406072 (0.028574) | 0.499222 (0.038070) |
| SVM-SMOTE (1:2) | 0.863329 (0.014990) | 0.448010 (0.038025) | 0.924944 (0.004640) | 0.457443 (0.032408) | 0.475272 (0.034807) | 0.440902 (0.037771) |
| SVM-SMOTE (1:3) | 0.870072 (0.014625) | 0.459616 (0.040201) | 0.927679 (0.004661) | 0.467105 (0.033892) | 0.495637 (0.038069) | 0.441680 (0.038077) |
| SVM-SMOTE (1:4) | 0.872144 (0.014412) | 0.464235 (0.040508) | 0.927623 (0.004491) | 0.452512 (0.035779) | 0.494922 (0.037153) | 0.416796 (0.037050) |
| SVM-SMOTE (1:5) | 0.877092 (0.013963) | 0.472865 (0.039980) | **0.930022 (0.004182)** | 0.460413 (0.034229) | 0.515414 (0.039555) | 0.416019 (0.037990) |


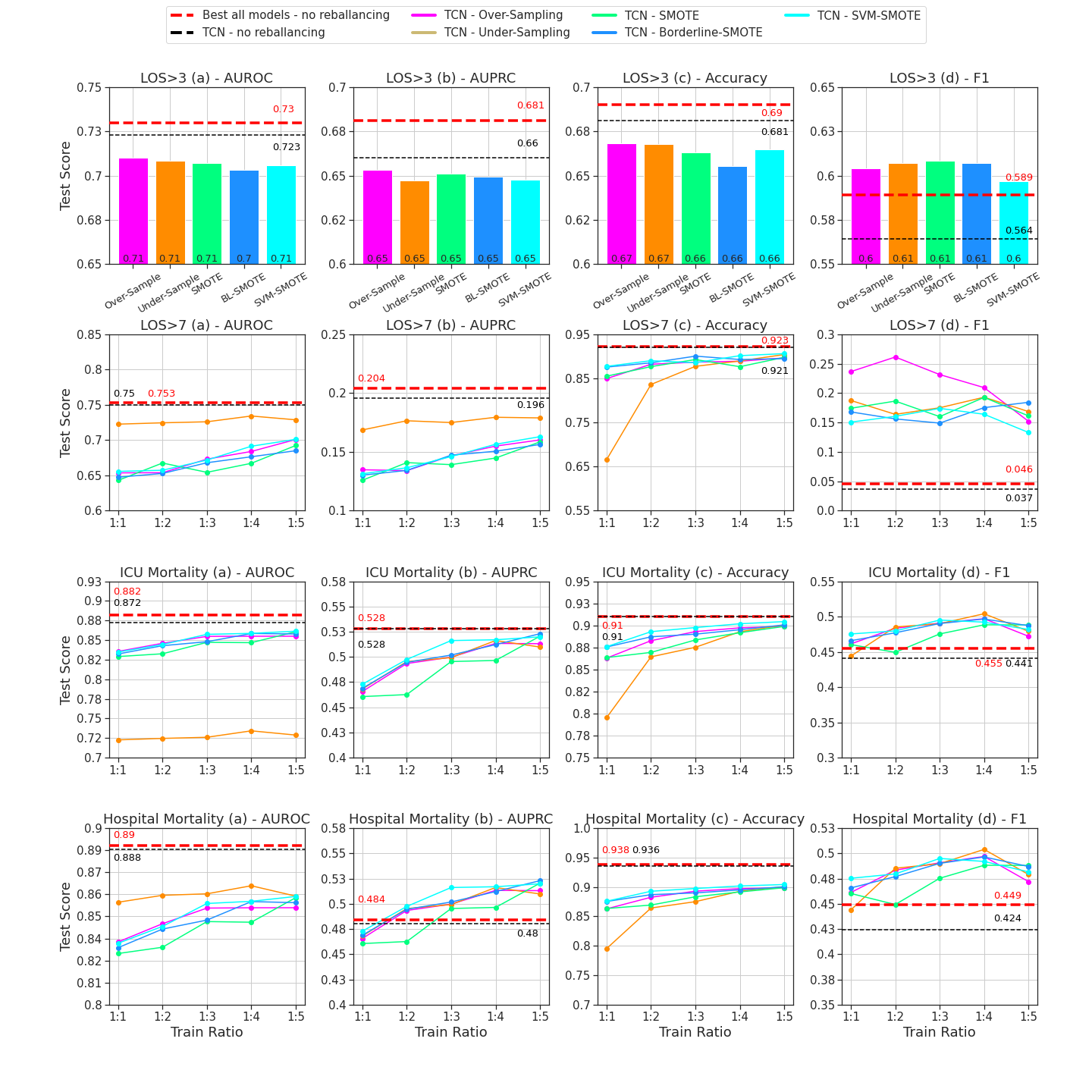


**Supplementary Figure 9:** Cross-validation rebalancing results figure for visual verification of consistency with hold-out counterpart

**Supplementary Appendix D - TCN Hyperparameters**

**Supplementary Table 4** - TCN hyperparameter grid search range

| **TCN Parameter** | **Description** | **Search Range** | **Optimal Value From Search** |
| --- | --- | --- | --- |
| Levels | number of hidden layers in the TCN network | [6, 15] | 12 |
| Kernel Size | width of sliding 1-d convolutional kernel | [2, 8] | 5 |
| Dilation Factor | exponential factor of dilation at each successive level of TCN | [2] | 2 |
| NHID | number of kernels at each level, kernel density. Also represents the density of the linear layer at the end of the model | [15,250] | 209 |
| Batch Size | number of training samples per back prop | 4,16,32,64,128,258 | 128 |
| Dropout Rate | node dropout rate | [0.5,0.99] | 0.8561711076 |
| Learning Rate | node learning rate | [1e-5,5e-4] | los>3=7e-5; los>7=8.5e-5, icu_mort=6.5e-5, hosp_mort=7e-5 |
| Learning Rate Degradation | determines whether learning rate will ramp up or decay exponentially. lr = learning_rate * (lr_deg) ** epoch # lr_deg 0 < x < inf | [0,3.0] | 1.497957374 |
| Loss Function | type of loss function used for classification. Defaults to MSE loss for regression | BCE_logits_loss, hinge_loss | BCE_logits_loss |
| Clamp Value | node weight clamp value | [0.5,3.5] | 1.377311954 |
| AMS Grad | flag whether or not to use AMS Grad option for pytorch Adam optimizer | [0,1] | 1 |
| Input Channels | number of features * hours | 104 (just values, no mask or time since meas.), 312 | 312 |
| Output Channels | n_classes, output classes | 1,2 | 2 |
| Early Stop Fraction | training data reserved for early stopping of TCN training | 0.05 | 0.05 |
| Patience | number of epochs to wait until early stopping is engaged | [3,10] | 3 (for speed) |
| SEED | randomization seed for numpy, pytorch, pandas, python.random | 1 | 1 (for consistency) |
